# Photochemistry of an Anti-Bredt Olefin through the Lens of Multistate Multireference Quantum Chemistry

**DOI:** 10.1021/jacs.5c22985

**Published:** 2026-04-21

**Authors:** Meseret Simachew Bezabih, Filippo Sacchetta, Alejandro Blanco-González, Massimo Olivucci

**Affiliations:** † Department of Chemistry, 1888 Bowling Green State University, Bowling Green, Ohio 43403, United States; ‡ Dipartimento di Biotechnologie, Chimica e Farmacia, Università di Siena, Siena I-53100, Italy

## Abstract

In 2024, Garg and co-workers reported that norborn-1-enes, a class of *anti*-Bredt olefins, can be systematically prepared and trapped. This finding has prompted us to combine multistate, multiconfigurational quantum chemical gradients and multiscale modeling to simulate the light-induced dynamics and chemistry of norborn-1-ene in acetonitrile. The results predict the existence of an excited state intermediate with a unique electronic structure consisting of a zwitterion incorporating a nonclassical cationic moiety. A set of 200 room-temperature quantum-classical trajectories were propagated to show that such intermediate decay through a unique conical intersection leads to the simultaneous formation of a carbene and a diradical as primary photoproducts. A third zwitterionic photoproduct is instead predicted to have a transient existence. Thus, our simulation not only uncovers a new type of photochemical funnel but also points to novel chemistries only accessible when *anti*-Bredt olefins are prepared or trapped under illumination conditions.

## Graphical abstract

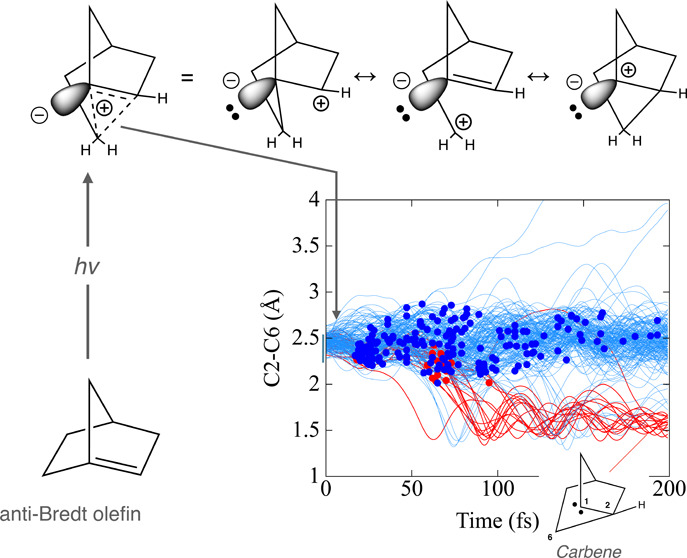

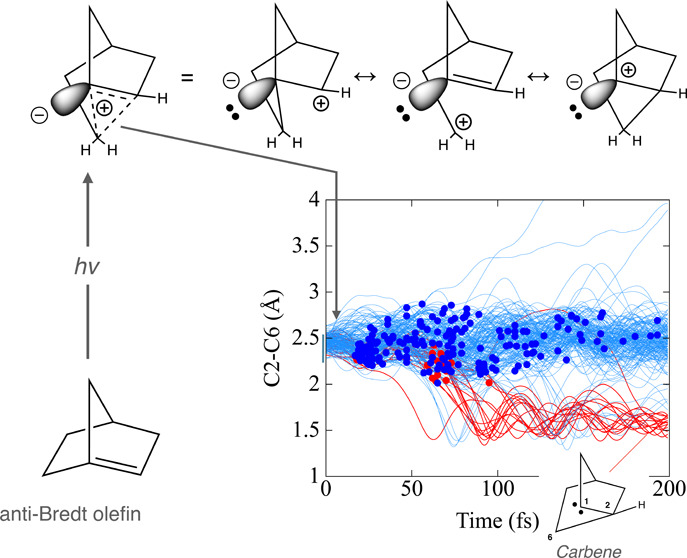

## Introduction

For more than a century, chemists have been fascinated by highly strained olefins.^1–3^ Bicyclic olefins with a bridgehead C=C bond are highly strained olefins.^4^ The strain comes from the distorted geometry of the bridgehead sp^2^ carbon atom, displaying large deviations from the expected planarity.^5–7^ Since Julius Bredt’s 1924 observation that pinanes and camphanes avoid bridgehead double bonds plus several failed synthesis attempts,^6–12^ the “Bredt’s rule” was announced stating that the formation of bridgehead olefins is not possible. However, in the late 20th century, chemists started to question Bredt’s rule, defining synthesis targets known as *anti*-Bredt olefins (ABOs).^4,13–27^ For instance, in the 1990s, Wiberg, Platz, and Eguchi’s groups took advantage of carbene rearrangements to generate different bridgehead olefins.^18–20^ More recently, in 2019, Wang and Ma used a base-mediated elimination to transiently generate a bicyclo[3.2.1]­oct-1-en-3-one intermediate.^28^ These works indicated that Bredt’s rule may be broken, thus establishing ABOs as synthetically accessible and, possibly, opening the path to novel chemistries. On the other hand, the achieved olefins featured relatively large rings (>5 membered rings) and less distorted sp^2^ carbons. It was thus argued that these systems could still conform to Bredt’s rule.^27^

In 2024, Garg and co-workers^5^ elegantly demonstrated that a set of norborn-1-ene (i.e., bicyclo[2.2.1]­hept-1-ene) derivatives featuring a formally sp^2^ bridgehead carbon shared by two 5-membered rings could be prepared in solution and lived long enough to be captured by anthracene via a 4π + 2π cycloaddition reaction. The successful synthesis of genuine ABOs prompts a comprehension of their electronic structure, without which it would not be possible to fully understand their stability and reactivity. When taking parent compound **1** as a reference (see Figure 1a top-left), it is evident that its structure must be characterized by a nearly broken π-bond resulting from a limited overlap between a pure and a hybridized p-orbital (see Figure 1a, top-right). In this situation, the π and π* molecular orbitals of the double bond become closer in energy, and consequently, the ground (S_0_) and excited (S_1_) singlet electronic states must become closer in energy and shift light absorption to longer wavelengths. Accordingly, the study of **1** requires quantum chemical methods capable of dealing with variable electronic structures since its geometrical distortions and closer electronic states may lead to mixed electronic characters including covalent, charge transfer, or diradical components. Furthermore, at certain geometrical deformations, the S_0_ and S_1_ states could become degenerate at a conical intersection (CoIn).

**Figure 1:**
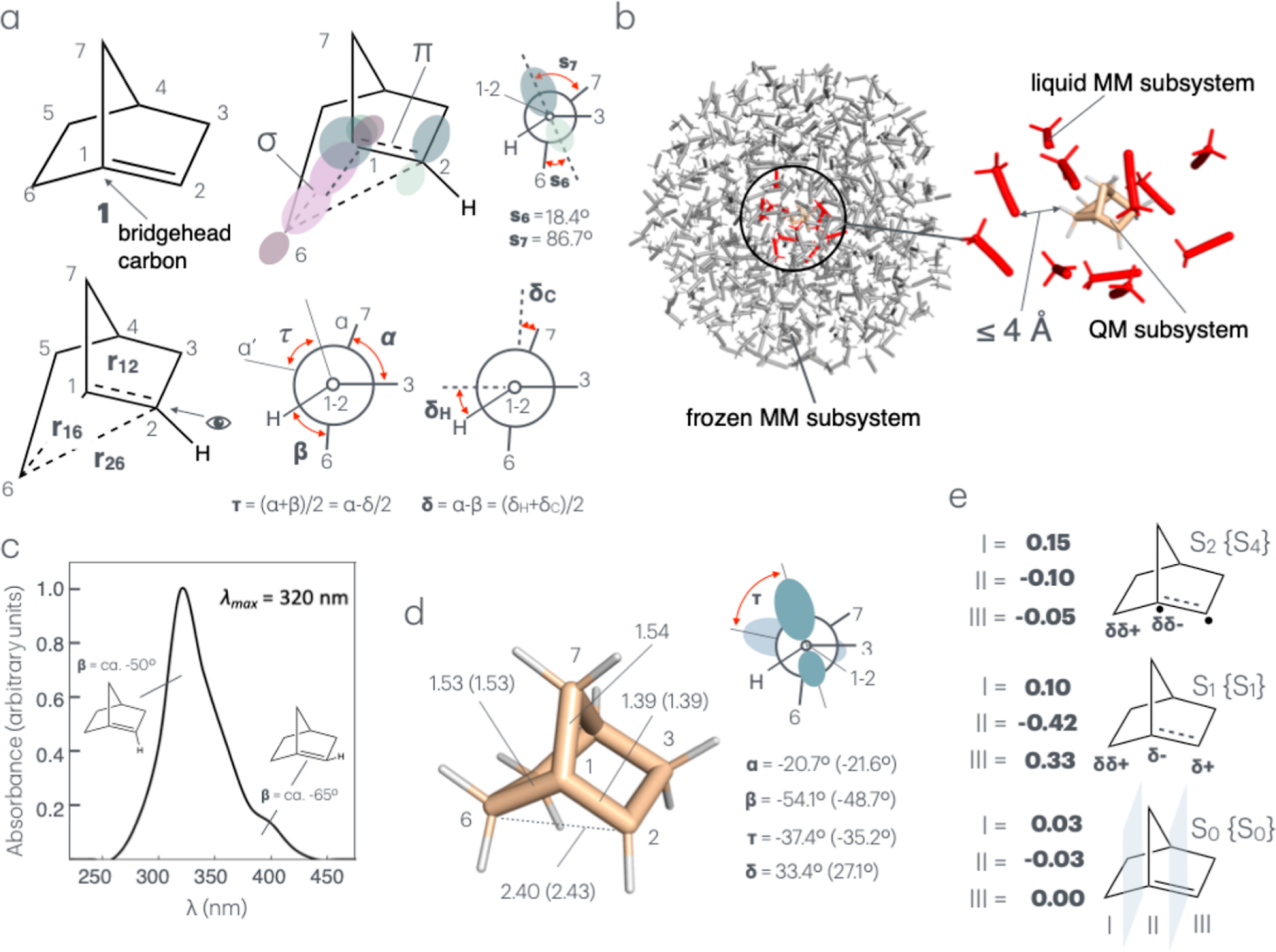
Norborn-1-ene model, active space, and equilibrium structure. (a) Atom numbering in parent norborn-1-ene **1** (top-left), definition of the active space orbitals (top-right), and bond lengths, bond angles, and dihedral angles (bottom) contributing to its reaction coordinates. The sp hybrid orbitals forming the molecular orbitals included in the active space are depicted in purple (σ-bond) and cyan (π-bond). The **s**_**6**_ and **s**_**7**_ dihedral angles inform on the approximate alignment of the C6–C1 and C7–C1 σ-bonds with the p-orbital on C2. (b) Structure of the QM/MM model of **1** in MeCN. The flexible solvent cavity is defined by the solvent molecules with at least one atom at *a* ≤ 4 Å distance from any atom of the solute (11 MeCN molecules). (c) Computed UV–vis absorption band at room temperature. The assignment of the band shape, via conformational analysis (e.g., through the distribution of the values of β), is also reported. (d) Computed S_0_ equilibrium structure of **1**. The values in parentheses correspond to the gas phase. (e) Mulliken charges for moieties I–III. Mulliken charges (δδ < δ), which have been found consistent with the reported values are confirmed by the corresponding Hirshfeld charges (see Table S4). The corrected state labeling in curly brackets reflects the presence of two low-lying Rydberg states below the valence state S_2_ (see Table S7).

The relatively recent development and implementation of multistate multiconfigurational (MSMC) complete active space (CAS) perturbation theories^29–32^ have provided a substantial improvement in the balanced treatment of ground and electronic excited states with electronically diverse or mixed characters. Furthermore, certain MSMC methods allow for a correct description of the electronic structure even in a situation of degeneracy or near degeneracy, as in regions surrounding a CoIn. One of these methods, available in the quantum chemistry package [Open]­Molcas,^33^ is called rotated multistate CAS second-order perturbation theory (RMS-CASPT2), and it is characterized by the absence of parameters in the multistate Hamiltonian with respect to the more popular extended-multistate CASPT2 (XMS-CASPT2) level.^34^ Most importantly for the present work, the RMS-CASPT2 potential energy gradients^34–38^ have become available, allowing the mapping of potential energy surfaces (PESs) as well as the propagation of on-the-fly classical and quantum-classical trajectories. These gradients can also be used within a multiscale model of the system under investigation to simulate reactions in solution or in a macromolecular cavity.

The reduced S_1_–S_0_ energy gap and potentially low-lying CoIn of **1** prompt strong interest in its photochemistry. In the present work, we focus on the multiscale quantum-mechanics molecular mechanics (QM/MM) study of the presently unknown room-temperature photochemistry of **1** in acetonitrile (MeCN). The QM/MM model is constructed using the [Open]­Molcas/Tinker interface.^39^ We describe **1** at the 3-root-state-average (SA3) RMS-CASPT2/aug-cc-pVDZ quantum mechanical (QM) level and solvent molecules described at the OPLS-aa^40^ molecular mechanics (MM) level. In the final model, the QM and MM subsystems interact sterically and electrostatically via electrostatic embedding. Our study has two complementary targets. The first is to use a thermalized molecular population characterized by 200 geometries and velocities to explore the electronic structure changes achieved after its photoexcitation and, ultimately, extract information about the S_1_ and S_0_ PES landscapes. The second is to predict the experimentally untested photoinduced dynamics of norborn-1-enes and their photochemistry and thermal stability. Note that while the use of the selected QM level of theory appears unavoidable when exploring unknown electronically excited-state processes, its high computational cost often prevents its use. However, the limited size of **1** as well as the assumption that the dominating reactive processes involves the π-bond and the C6–C1 σ-bond exclusively (i.e., the C6–C1 bond most effectively aligns with the p-orbital on carbon C2 when compared to the C7–C1 bond; see the **s**_**6**_ and **s**_**7**_ dihedral angle values in Figure 1a, top-right) makes our study affordable. Accordingly, the employed CAS is defined by four electrons residing in the four (π, π*, σ, and σ*) molecular orbitals (see Figure S1) resulting from the combination of the sp^n^ orbitals in the top-right of Figure 1a. The validity of the above assumption is supported by recomputing the energy and geometry of different stationary points with an expanded CAS also including the σ and σ* molecular orbitals of the weakly aligned C1–C7 bond (Figure 1a top-right).

Below, we show that quantum-classical trajectories driven by RMS-CASPT2/aug-cc-pVDZ gradients and nonadiabatic couplings predict the existence of a unique excited-state intermediate and CoIn stabilized by a homoaromatic interaction. Such a CoIn functions as a “trailhead” for the potential population of S_0_ relaxation channels featuring carbene, diradical, and zwitterionic characters.

## Results and Discussion

The QM/MM model of **1** in MeCN (see Figure 1b) is defined by a QM subsystem corresponding to the solute and a MM subsystem divided in a fixed (frozen) solvent shell incorporating a central drop of flexible (liquid) solvent molecules. Such a model is employed to simulate the system room-temperature Boltzmann distribution via our in-house computational protocol, which has previously been optimized and standardized for pyrimidine nucleobase^35^ and molecular motors,^41–44^ yielding 200 uncorrelated snapshots. These are then used to (i) compute the UV–vis absorption band by calculating, for each snapshot, the S_1_–S_0_ energy gap and the corresponding oscillator strengths and by convoluting the results (see Scheme S1) and (ii) provide the initial conditions for propagating 200 quantum-classical trajectories (i.e., Tully’s fewest-switches surface-hopping trajectories^45^ with a decoherence correction^46^ value of 0.1 a.u.) simulating the system photoinduced population dynamics. A model representing the S_0_ equilibrium geometry of **1** in solution is constructed by extracting the snapshot (called FC snapshot in the following) with the excitation energy closest to the computed λ_max_ of the absorption band (see Figure 1c) and relaxing its geometry to S_0_. The computed S_1_-S_0_ excitation energy of **1** in MeCN has been found to correspond to a 316.5 nm photon, with an oscillator strength value of 0.135, and is reported in Table S1. Below, we discuss the results i and ii and show that the π → π* photoexcitation prompts C6–C1 bond expansion and shifting, leading to homoaromatic stabilization of the S_1_ state and to a S_1_/S_0_ CoIn. All of the computational details are provided in the Methods section of the Supporting Information.

### UV–Vis Absorption Indicates that S_1_ is the Spectroscopic State of **1**

The calculated absorption band of **1** in MeCN is shown in Figure 1c. The λ_max_ value obtained at the SA3-RMS-CASPT2/aug-cc-pVDZ level of theory is 320 nm and corresponds to the S_0_ to S_1_ vertical transition. The SA3-RMS-CASPT2/aug-cc-pVDZ optimized geometry of the solvated and isolated solute is reported in Figure 1d which shows that, for isolated **1**, the relevant parameters are close to the DFT-optimized geometry (see Table S2 for the comparison of dihedral angles analysis) reported in the literature.^5^

The Mulliken charge analysis of the S_0_ equilibrium structure (see Figure 1e) shows that the vertical S_1_ state displays a charge transfer (i.e., zwitterionic) character relative to S_0_ and has an oscillator strength (*f* = 0.135) consistent with dominating π → π* single excitation localized on the geometrically distorted C1=C2 bond. S_1_ is therefore identified as the spectroscopic state. Due to its diradical character, the S_0_-to-S_2_ vertical transition is found at a higher excitation energy and features a negligible oscillator strength (*f* = 0.054). As detailed in the Supporting Information, while S_1_ and S_2_ are valence states, two Rydberg states lower than S_2_ are identified by expanding the CAS to include one 3S and one 3P diffuse orbitals of the same basis set.^47^ However, while S_2_ must be reassigned to S_4_ (See Figure 1e), its energy and diradical character are substantially unchanged. The fact that, in contrast with unstrained olefins,^48,49^ S_1_ is located below the Rydberg states supports the use of the selected four-electron four-orbital CAS for PES mapping and quantum-classical trajectory computations. This is reinforced by the fact that the energy gap among S_1_, the Rydberg states, and the diradical state increases after geometrical relaxation along the S_1_ PES.

### Quantum-Classical Dynamics Points to an Ultrafast Excited-State Decay

The photoinduced dynamics of **1** in MeCN is characterized by S_1_-to-S_0_ hopping events that are completed within 200 fs (no S_0_-to-S_1_ back-hopping events have been observed). These are marked by black circles in Figure 2a, where we report, for the entire room-temperature population, the progression of the C1–C2 stretch initiated by S_0_ to S_1_ excitation. The earliest hop occurs at ca. 20 fs, while the last is detected at ca. 200 fs during a relatively coherent oscillatory nuclear motion. This leads to an average S_1_ lifetime of the order of 75 fs (see Figure S2). Such behavior indicates the existence of a barrierless path on the S_1_ PES, leading to an S_1_/S_0_ intersection space (IS). Decay at any point of the IS (i.e., at a CoIn point) leads either to internal conversion (see movie Reactant.mp4) or to the formation of photoproducts. In the following, these events are marked by blue circles and red circles, respectively. The generation of a photoproduct (i.e., a carbene) is seen in Figure 2b, where we report on the progression of a selected trajectory. The top-left panel of the figure shows that a point of reactive hop is reached after ca. 25 fs. The top-right panel shows that the double bond length expands and acquires the typical bond length of a C-C single bond (ca. 1.54 Å). In contrast, the bottom panels display simultaneous breaking of the C1–C6 bond and formation of the C2–C6 bond to yield a carbenoid structure produced via a suprafacial [1,2] sigmatropic rearrangement (see movie Carbene.mp4).

**Figure 2:**
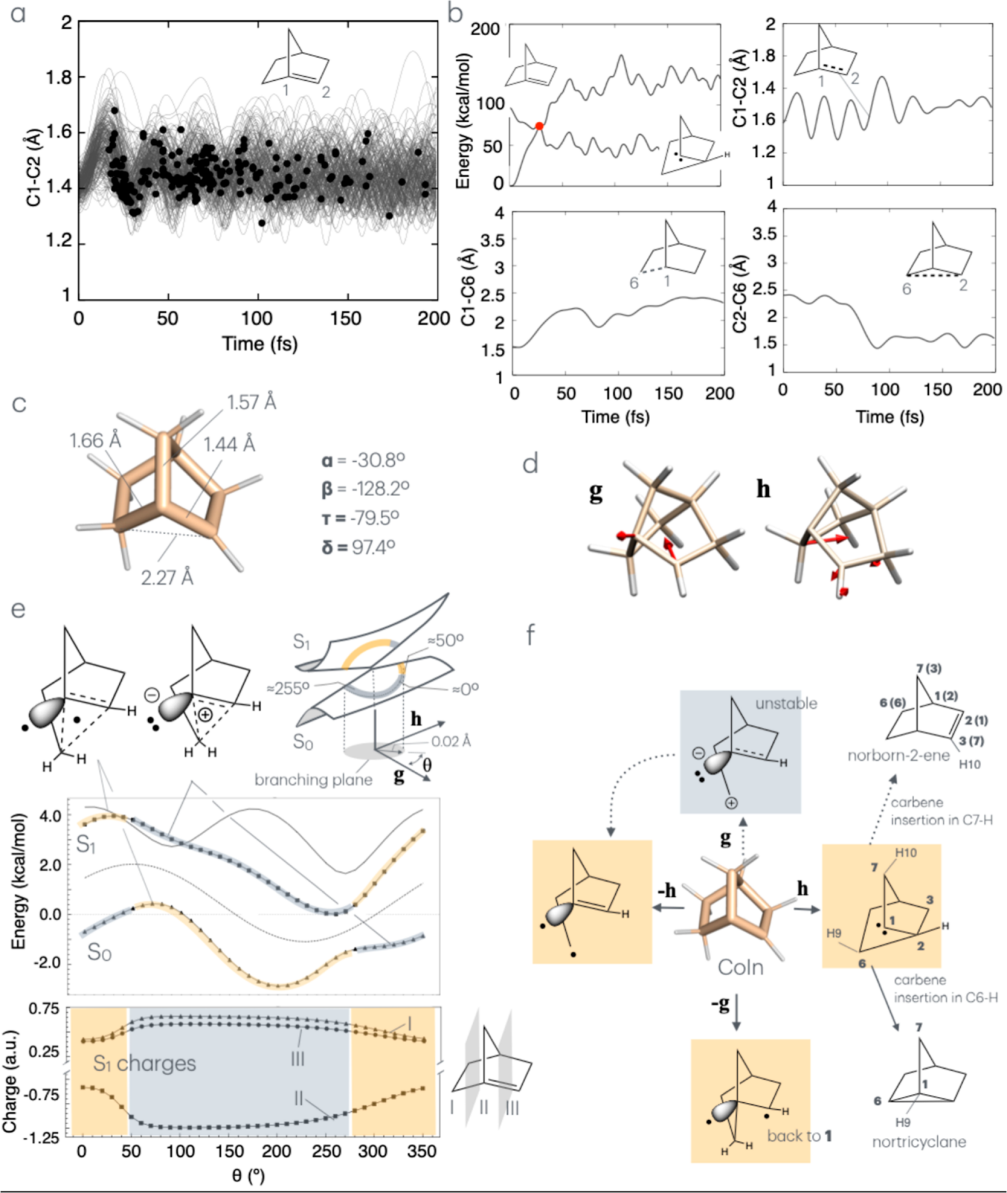
The conical intersection of norborn-1-ene. (a) C1–C2 length progression along the quantum-classical trajectories representing the photoinduced dynamics of **1**. The hopping points (i.e., the points of decay from S_1_ to S_0_) are marked with a black circle. (b) Analysis of a single reactive (hopping point is marked with a red circle) trajectory. The S_1_ and S_0_ potential energy change is given in the top-left panel. The remaining panels give the progression along relevant geometrical coordinates. (c) Equilibrium structure of a representative minimum energy CoIn derived from the hop point in panel b. (d) **g** and **h** vectors of the CoIn in point c. (e) Branching plane analysis. The yellow color corresponds to a dominating covalent/diradical character, while the gray color corresponds to a dominating charge transfer character (see schematic representation at the top-left). (f) Branching plane diagram displaying the predicted primary photoproducts and possible final products. The two coexisting electronic characters of the CoIn allow for the formation of either diradical/covalent or zwitterionic character (yellow color and gray color as in part (e). Full and dotted arrows indicate products that are detected or undetected via quantum-classical trajectory calculations, respectively (see text). The numbering in parentheses in the potential norborn-2-ene secondary product refers to the parent carbene.

As discussed below, all trajectories in Figure 2a can be analyzed on the basis of dihedral angles such as α, β, τ, and δ (see Figure 1a), which provide information on the carbon pyramidalization and sp-orbital overlap across the C1–C2 bond. For instance, the evolution of β demonstrates that the hopping occurs in the region of an IS segment featuring a β value falling in the 50°–200° range and therefore is distant from the values associated with the initial conditions (see the Boltzmann sampling distribution of dihedral angles in Figure S3). In contrast, at the hopping points, α displays values falling in a ca. 0°–50° range and, therefore, not far from the initial conditions.

The dynamics of **1** also reveal coherent (i.e., phased) nuclear motion of the molecular population along several geometrical parameters such as bond lengths and torsion angles. For instance, it is apparent from inspection of Figure 2a that the C1–C2 bond length displays conserved oscillations with a period of ca. 30 fs that partially lose coherence after 100 fs and, therefore, after a significant part of the population has been transferred (hopped) to S_0_ leading to either internal conversion or photoproduct formation. This behavior has previously been reported for molecular switches^50^ and visual pigments.^51^

### The CoIn of **1** is a Crossing between a Homoaromatic Zwitterionic Excited State and a Delocalized Diradical Ground State

In Figure 2c, we display the structure of a representative minimum-energy CoIn of **1** in MeCN (i.e., a local minimum of the IS). This was calculated via minimum energy conical intersection optimization^52,53^ starting from the structure of the early (i.e., occurring) hopping point in Figure 2b (see the red circle in the top-left panel). Consistency with the S_0_ equilibrium geometry in solution of Figure 1d was ensured by starting the trajectory from the FC-snapshot defined above. The mapping of the surrounding S_1_ and S_0_ PESs along the two-dimensional branching plane defined by the gradient difference vector **g** and derivative coupling vector **h** (see Figures 2d and S4) reveals a slightly sloped topography (see Figures 2e and S4) with the S_1_ exit pointing in the direction of an S_1_ intermediate.^34,54,55^ The sloped nature of the CoIn is confirmed by the value of relative tilt,^56–58^ as well as by the S_1_ and S_0_ energy profiles computed along a circular cross section of the branching plane (see center of Figure 2e), where the local S_1_ and S_0_ relaxation (exit) channels are located at θ of ca. 250° and 200°, respectively. At the bottom of Figure 2e, we instead provide information on the change in fractional Mulliken charges of fragments I–III (i.e., same as in Figure 1e) along the cross section. These show that the CoIn electronic structure is characterized by degenerate diradical (yellow color) and charge transfer (gray color) characters that, consistently with the presence of a geometric phase,^59^ dominate different parts of the circular energy profiles and exchange role when switching from S_1_ to S_0_. This interpretation, based on the charge analysis (see Tables S4), has been confirmed via Mayer’s free valence analysis whose results are reported in Table S5 (i.e., the analysis shows that in the part of the loop with charge transfer character, the C1 carbon has approximately three bonds and no free valences; in contrast, in the diradical part, it has three bonds and one free valence). As we will discuss below, the two degenerate electronic states at a CoIn (see the top-left of Figure 2e) are interpreted as a zwitterionic structure characterized by a moiety featuring a delocalized homoaromatic cation and a diradical structure, where the same moiety features a delocalized radical.

By combining the information on the CoIn geometry, the direction of **g** and **h**, cross-section energies, and electronic characters, it is possible to infer the S_0_ relaxation paths *populated* after S_1_ decay (i.e., after the hop from S_1_ to S_0_). These are given in the diagram in Figure 2f. It is apparent that a relaxation along **g** leads to either a zwitterionic (positive direction, gray color) or diradical (negative direction, yellow color) intermediate. The relaxation along **h** would instead lead to a carbene or a distinct diradical intermediate in the positive and negative directions, respectively. Note that the singlet diradical character (given the extremely short reaction time scale, we do not discuss the possibility of triplet diradical formation here) becomes covalent when the two unpair electrons couple to form a bond. Thus, the diradical produced by relaxation along **–g** is likely to be a precursor of **1**. The geometrical structures of the optimized geometries on S_0_, S_1_, CoIn, and the photoproducts are provided in Figure S5.

### Predicted Photoproducts and Decay Mechanism

Quantum-classical trajectory analyses of the type seen in Figure 2a,b allow prediction of which reaction channel in Figure 2f is populated. First, as shown in Figure 3a–c, all trajectories except five describe a relaxation from the Franck–Condon (FC) region dominated by the simultaneous expansion of the C1–C2 and C1–C6 bonds to reach a low energy region of the S_1_ PES close to the CoIn. Indeed, S_1_ geometry optimization starting from the CoIn of Figure 2c yielded the S_1_ energy minimum, an S_1_ intermediate, of Figure 3d located about 1.2 kcal/mol below the CoIn. This process is schematically represented by the S_1_ relaxation of Figure 3e. The results of charge analysis (see Figure 2e) applied to the S_1_ intermediate point to an electronic structure consistent with that of a unique intramolecular charge transfer system featuring a 3-center homoaromatic cationic moiety (a Olah’s nonclassical cation^60^) incorporating an orthogonal carbanionic center at C1. In valence-bond language, such a cation is described by the combination of three resonance formulas each associated with a circulating position (see dashed triangle) of the positive change and of the two electrons necessary for aromatic stabilization. It is important to mention that the formation of the homoaromatic cation from the FC region must occur through “anchimeric assistance” which stabilizes the system along the S_1_ relaxation, helping the π-bond breaking process. It is also important to note that the cationic moiety is in its “ground state” even if the entire molecule resides in S_1_. Note that at the equilibrium geometry of the homoaromatic S_1_ intermediate, the S_0_ electronic structure corresponds to a delocalized diradical with one electron delocalized along the same 3-center moiety, hosting the cation in S_1_ and the other electron isolated on an orthogonal orbital at C1. These S_1_ and S_0_ electronic structures must correlate with the degenerate and electronic structures at CoIn (see Figure 2e).

**Figure 3:**
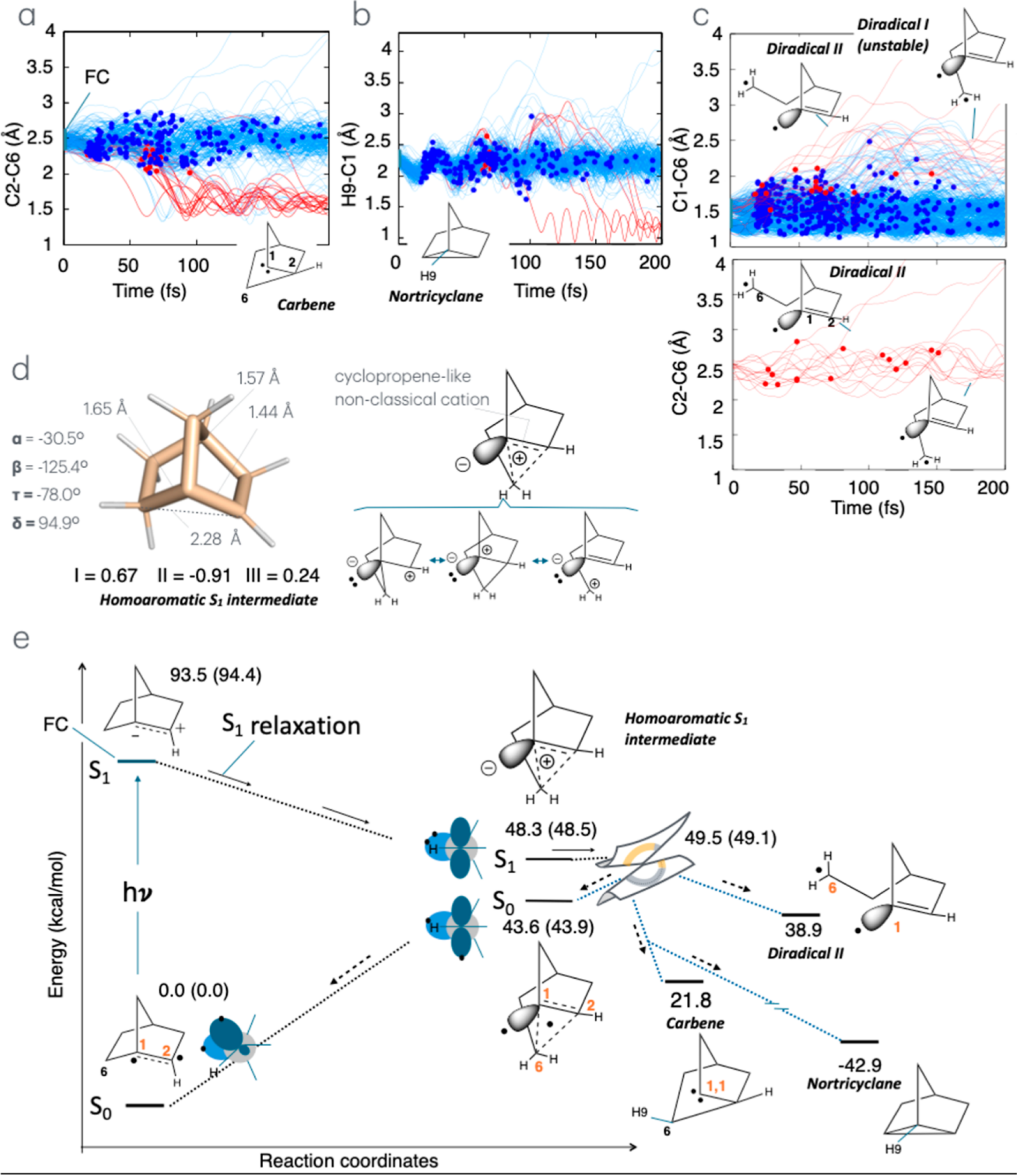
Nonadiabatic molecular dynamics and reaction mechanism of norborn-1-ene. (a) Time evolution of the C2–C6 bond lengths of **1** during the excited-state (S_1_) dynamics responsible for the formation of carbene (red trajectories), also referred to as reactive trajectories. The circles filed with red and blue colors on each trajectory always correspond to hopping points from S_1_ to S_0_ in the IS region. The hopping points filed with red color along with red-colored trajectories lead to the formation of carbene. (b) Time evolution of the H9–C1 bond length of **1**. Red trajectories are reactive, i.e., responsible for the formation of the nortricyclane photoproduct, while blue trajectories are always nonreactive. (c) Time evolution of the C1–C6 and C2–C6 bond lengths of **1**. The panels at the top and bottom are used to determine whether those trajectories represent real diradicals. (d) Geometrical parameters and fragment (see Figure 1e) charge distribution for the S_1_ potential energy minimum of **1** with a schematic representation of its electronic structure and the resonance formula. (e) Schematic S_0_ and S_1_ potential energy profiles along the photochemical reaction path of **1** and related mechanistic information. There are two primary photoproducts: carbene and diradical generated after decay in the CoIn region, along with one formally secondary photoproduct, nortricyclane. The relative energy value (kcal/mol) are given for the geometrically optimized structure featuring consistent solvent cavities. The values in parentheses refer to the energies computed with an expanded active space incorporating the C1–C7 σ and σ* orbitals (see Table S3).

We now discuss the relationship between the CoIn properties and the reactivity of distinct quantum-classical trajectories of **1**. This is done (see Figure 3a–c) (i) through geometrical and charge analyses, (ii) by predicting the formation of primary and secondary photoproducts in S_0_, and (iii) by estimating their photochemical quantum efficiency (i.e., the ratio between the number of product molecules generated and the number of photoexcited reactant molecules, Φ_photochem_). In fact, in Figure 3a, we display a set of trajectories, leading to the formation of a σ-bond between C6 and C2 whose distance decreases from ca. 2.5 to 1.5 Å. This process can only occur during the formation of a carbene intermediate. Such a carbene can be produce by exiting the CoIn along the region of the S_0_ PES with diradical character (i.e., along the yellow region of the S_0_ energy profile of Figure 2e). The formation of a singlet carbene on the C1 center is confirmed by geometry optimization starting from the last point of a selected trajectory (the same displayed in Figure 2b). This has been confirmed by successive charge such as Mulliken and atomic dipole-corrected Hirshfeld atomic charge (ADCH) analysis (see Table S4) as well as free valence and bond order via Mayer’s analysis (see Tables S5 and S6). These results have been used to mark the carbene-forming trajectories in red (Figure 3a).

In Figure 3b, we show that the trajectories also predict the ultrafast formation of a photostable secondary product, possibly originating from the transient carbene. Indeed, we show that geometrical analysis detects nortricyclane (see movie Nortricyclane.mp4), a tricyclic hydrocarbon that, formally, would form through the insertion of the carbene center at C1 in the C6–H9 bond of **1**, yielding the observed short C1–H9 distance (see the C6–H carbene branch in Figure 2f). Note that while nortricyclane may be considered a secondary photoproduct, the time scale of its formation combined with geometrical analysis suggests that it could be generated without the complete formation of a carbene intermediate though a substantially concerted process. On the other hand, this appears to be an inefficient event as only 5 trajectories led to nortricyclane. Finally, no trajectory led to the conventional norborn-2-ene via a process that could be thought of as the insertion of the same carbene center at C1 in the C7–H10 bond (see the C7–H carbene branch in Figure 2f). Thus, with the limitation associated with the analysis of 200 trajectories, we estimate Φ_photochem_ values of 14% and 3% for carbene and nortricyclane, respectively.

In Figure 3c, we also predict the formation of a diradical intermediate in two distinct conformations called Diradical I (see movie Diradical-I.mp4) and Diradical II (see movie Diradical-II.mp4). These are generated via, again, relaxation from the diradical region (yellow color in Figure 2e) of the S_0_ PES surrounding the CoIn. Geometrically, they are detected by observing a simultaneous increase of the C6–C1 and C6–C2 distances together with a concurrent charge and Mayer’s analyses (for the Mayer’s results, see Tables S5 and S6) that reveal a neutral C1 center with three valences and one free valence. A stable S_0_ energy minimum could only be found for Diradical II featuring a conformation, where the CH_2_ radical center at C6 is distant from the radical center at C1 and, thus, live longer (the formation of Diradical II is predicted with a Φ_photochem_ value of 8%). In contrast, S_0_ geometrical relaxation starting from different trajectory snapshots of Diradical I, always lead to radical recoupling and reconstitution of **1** consistent with a singlet diradical species. A direct reconstitution of **1** is instead observed when the C6–C1 distance contracts and the C6–C2 distance expands immediately after the trajectory has decayed.

The formation of two distinct photoproducts simultaneously with the reconstitution of **1** is explained by the electronic structure of the diradical region of the S_0_ PES surrounding the CoIn. As mentioned above, such a region is characterized by the same delocalized diradical seen in the S_0_ state of the homoaromatic S_1_ intermediate of Figure 3e. During S_0_ relaxation, the delocalized electron will localize in one of the carbon atoms of the 3-center moiety marked by the red numbers 1, 2, and 6 (i.e., C6, C1, or C2 indicated by the three red numbers). Localization of both electrons in C1 (positions 1,1) yields a carbene, localization on C6, C1 (positions 1,6) leads to a diradical, and localization on C1, C2 (positions 1,2) leads to the reconstitution of the bridgehead double-bond of **1**.

Note that none of the computed trajectory leads to a zwitterionic intermediate of the kind indicated by the positive direction of **g** in Figure 2f. As detailed in Figures S6 and S7 of the Supporting Information, the possible production of a S_0_ zwitterion incorporating a homoaromatic nonclassical cation is investigated by first mapping a more extended region of the S_0_ PES with zwitterionic character (e.g., the gray area in Figure 2e corresponding to the zwitterionic side of the S_0_ PES centered at CoIn) and successively showing that no energy minimum could be located via geometrical relaxation starting in that region. We conclude that, consistent with trajectory analyses, the formation of a stable zwitterionic photoproduct is unlikely due to back charge transfer along the S_0_ trajectory segments initiating in that region.

The results described above are collected in Figure 3e in a mechanistic picture, also including information on the thermal stability of **1**. In other words, the employed level of theory predicts that a barrier higher than 17.7 kcal/mol must be overcome to “decompose” **1** via the formation of the detected carbene. Note that the potential energy values displayed in the scheme refer to the single solvation shell for the representative CoIn discussed above. Therefore, they must be considered approximate/indicative. As detailed in the Supporting Information, these energy values appear to be insensitive to expansion of the CAS to include the C1–C7 bond orbitals. In fact, while the distinct homologue homoaromatic S_1_ intermediate and CoIn describing the breaking of the C1–C7 bond exist, these are located ca. 8 and 18 kcal/mol higher in energy than the corresponding C1–C6 structures.

## Conclusions

The availability of gradients for expensive, but close to quantitative, MSMC calculations has opened the path to the accurate study of chemical processes in which different electronic states mix or play an equivalent role. More specifically, by using such methods, the dynamic emergence of covalent/diradical and zwitterionic characters can be described in a balanced way and with an accuracy not available earlier by using quantum-classical trajectories. This is especially useful in the study of reactive intermediates and, most relevantly, electronically excited states. Above, we have shown that by using RMS-CASPT2 gradients and a QM/MM model, it has been possible to predict that the near-UV (ca. 320 nm) photoexcitation of an ABO olefin produces, in solution and in less than hundred femtoseconds (i.e., faster than typical diffusion time scales), a unique S_1_ zwitterionic intermediate incorporating a nonclassical carbocation. We have also shown that the S_0_ carbene intermediate, which has been experimentally detected in bridgehead olefins with larger rings,^61^ is a primary photoproduct originating from one of the resonance formulas of the zwitterionic S_1_ state upon decay toward the diradical side of the CoIn S_0_ PES. In contrast, we showed that the diradical originating from the other resonance formulas is produced less efficiently. Similarly, the S_0_ zwitterionic intermediate, that would be adiabatically related to the homoaromatic S_1_ intermediate, could not be detected and must only have a transient existence.

Our finding, provide a basis for the experimental investigation of the photochemistry of differently ABO olefins in terms of a unique and, to the best of our knowledge, novel excited-state intermediate and nearby CoIn (a photochemical funnel), where three different electronic characters coexist! In principle, the stabilization of one of these characters through substituent, solvent, or caging effects could yield different selectivities and, therefore, be useful in achieving different synthesis targets (e.g., like in the case of nortricyclane).

## Supplementary Material







## Notes

Associated movies for the reactant (Reactant.mp4) and products (Carbene.mp4, Nortricyclane.mp4, Diradical-I.mp4, and Diradical-II.mp4) reported in Figure 3 (ZIP)
Snapshots that contain velocities and coordinate along with the KEY and OPLS_aa.prm (ZIP)
Details of the computational method, vertical excitation energies, branching plane, singlet excited state lifetime, geometry and electronic structure analysis such as charge, free valence, and bond order, and optimized Cartesian coordinates (PDF)
