# Supplementary material for: Photochemistry of an Anti-Bredt Olefin through the Lens of Multistate Multireference Quantum Chemistry
Source: J Am Chem Soc. 2026 Apr 21;148(17):17736–44. doi: 10.1021/jacs.5c22985 (PMC13154192; doi:10.1021/jacs.5c22985)
Supplement: Supplementary file 3 [file ja5c22985_si_003.pdf]

Supporting Information:

Photochemistry of an Anti-Bredt Olefin through the Lens of Multistate Multireference Quantum Chemistry

Meseret Simachew Bezabih,[†] Filippo Sacchetta,[‡] Alejandro Blanco-González,[†] Massimo Olivucci[†,‡,*]

[†]*Department of Chemistry, Bowling Green State University, Bowling Green, Ohio 43403, United States*
[‡]*Dipartimento di Biotechnologie, Chimica e Farmacia, Università di Siena, I-53100, Siena, Italy*

Email: molvuci@bgsu.edu

Table of Contents

1. Methods 2
   1.1 Details of QM/MM Model Preparation 2
   1.2 Active Space Orbitals Selection 4
2 Excited State Lifetime 5
3 Boltzmann Sampling Distribution 6
4 Geometrical and Electronic Structure Analyses 6
   4.1 CoIn Topography along the Branching Plane 6
   4.1 Molecular Geometry Analysis 7
   4.2 Electronic Structure (Bonding) Analysis 9
   4.4. Is S_2 a Rydberg or valence state? 11
   4.5. Extended CoIn analysis and zwitterionic character 12
5 Cartesian Coordinates 14
7 References 22

1. Methods

1.1 Details of QM/MM Model Preparation

The initial step of this study was the preparation of the norborn-1-ene (**1**) molecule *in vacuo*, followed by optimization at the second-order Møller-Plesset level of theory using the 6-31G* Pople basis set (MP2/6-31G*)[1,2] with implicit MeCN as the solvent under the Polarizable Continuous Model (PCM)[3] implemented in the GAUSSIAN quantum chemistry software package.[4] Geometrically optimized **1** was then embedded in the center of a cubic box measuring 60 x 60 x 60 Å, containing 523 MeCN solvent molecules under the periodic boundary condition (PBC) model. The solute **1** (QM) was kept frozen, while the solvent (MM) subsystem was minimized at the molecular mechanics (MM) level using the conjugate-gradient method. The nuclear charges of the QM atoms were calculated using the Second Order Electrostatic Potential Fitting (ESPF) method at the MP2/6-31G* level. The bonding and van der Waals parameters were taken from the OPLS-AA force field.[5–8] After energy minimization, the system was relaxed for 5000 ps during a molecular dynamics (MD) simulation under the isothermal-isobaric (NPT) ensemble set at 1 atm pressure and 298 K temperature using the GROMACS MD software package.[9] During the first 300 ps of simulation, the system gradually thermalized at room temperature, then equilibrated for the next 700 ps, followed by relaxation for an additional 4000 ps. From this dynamic simulation step, referred to as the production step, 200 snapshots (uncorrelated momentum and configurations) were extracted every 10 ps, involving a 20.0 Å solvent layer used for the hybrid quantum mechanics/molecular mechanics (QM/MM) MD simulation. The solvent molecules located within ≤4.0 Å of any atoms from the QM molecule were extracted and treated at the MM level; these were allowed to move during QM/MM MD simulations, while the remaining solvent molecules were kept frozen. Finally, the QM/MM equilibration was performed at the MP2/aug-cc-pVDZ[10] level of theory for 200 fs, followed by an additional 200 fs at the SA3 RMS-CASPT2/aug-cc-pVDZ[11] level of theory, where SA3 indicates the number of roots included in the state averaging. At this point, the geometries were fully equilibrated and ready for a Boltzmann-like thermal population to perform calculations in the electronic ground state for the absorption spectrum and in the excited state (S_1) for quantum-classical dynamics simulations at the SA3 RMS-CASPT2/aug-cc-pVDZ level of theory, respectively. The ground state dynamics equilibration was performed at a time step of 1 fs or 41.3 a.u. In the following scheme, we present a summarizing graphical representation of the

computational protocol. In the present report, all electronic calculations, including quantum-classical trajectory simulation, in solution were performed using [Open]Molcas-v24.06/Tinker software[12,13] enabling a Tully surface hopping[14] algorithm with a decoherence correction value of 0.1 a.u. Throughout our calculations under the &CASPT2 block of [Open]Molcas input, we employed an IPEA and Imaginary values of 0.00 and 0.20, respectively. In the same block, we made sure that a "grdt" keyword was enabled, which is an important keyword in [Open]Molcas[13] to ensure analytical nuclear gradients are enabled in the calculation.

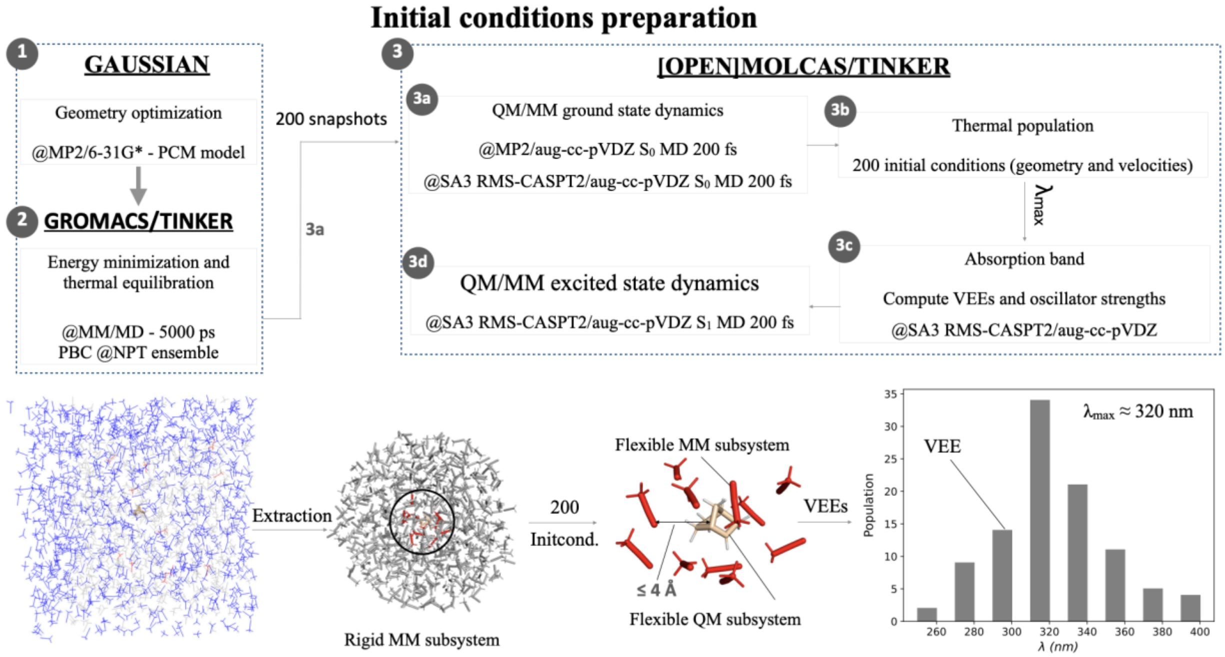

Scheme S1. Graphical overview of the computational protocol used in the present study. Upper panel (1-3): shorthand summary of preparing initial conditions for computing the absorption spectrum as well as S_1 quantum-classical dynamics. Bottom panel: periodic boundary condition (PBC) in a cubic box with a size of 60 x 60 x 60 Å that contains the QM and MM subsystems (bottom left). Frozen spherical shell of the QM/MM subsystem with the MM subsystem treated as rigid (gray), the MM subsystem treated as flexible (red), and the QM subsystem (tints) (middle left). Flexible QM/MM subsystem where **1** is treated as QM (C = tints and H = white), while all the surrounding 11 MeCN solvent molecules are treated as MM (red) (middle right). The VEEs of the stick model in nm (bottom right).

1.2 Active Space Orbitals Selection

In the present work, the complete active spaces for the electronic structure calculations, as well as for the quantum-classical trajectory simulations, were constructed with 4 electrons in 4 molecular orbitals (CAS(4,4)), comprising σ, σ*, π, and π* orbitals, where the σ and σ* orbitals correspond to C1-C6 and the π and π* orbitals correspond to C1=C2, respectively (see Figure S3 for atom indexing). In addition, we expanded the active space orbitals to CAS(6,6) to show that CAS(4,4) is sufficient for our study. In the expanded CAS(6,6), we incorporated the **σ** orbitals of the single bonds C1-C6 and C1-C7 adjacent to the C1=C2 double bond. In Figure S1, we show a plot of the molecular orbitals for both active space orbitals.

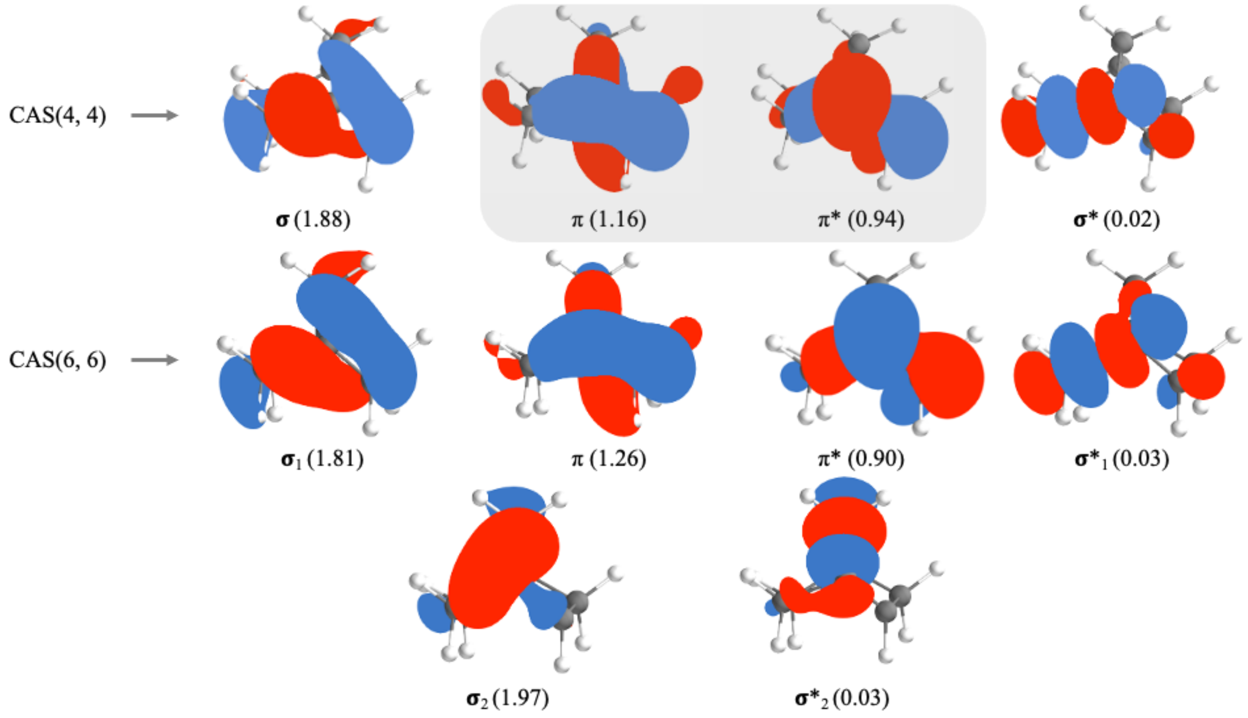

Figure S1. Complete active space molecular orbitals. Isosurface plots of the molecular orbitals used in the electronic structure calculation and analysis of **1**. The orbitals shown in the top panel are obtained with the CAS(4,4) space, and those in the bottom panel are obtained using the CAS(6,6) space. The highlighted molecular orbitals in the gray box (middle) emphasize the frontier molecular orbitals (FMO). The numbers right below each orbital are associated with the occupation numbers at the computed S_0 equilibrium geometry of **1** in MeCN (see S_0-min below). Orbitals were plotted using Pegamoid software with an isosurface value of 0.05.

Table S1. Vertical excitation energies and oscillator strengths in MeCN. The vertical excitation energies and oscillator strengths of the valence states S_0, S_1, and S_2 are computed using the aug-cc-pVDZ and 6-31+G* basis sets at the SA3 RMS-CASPT2 level of theory.

	SA3 RMS-CASPT2/aug-cc-pVDZ			SA3 RMS-CASPT2/6-31G*		
	VEE (kcal/mol)	VEE (nm)	*f*	VEE (kcal/mol	VEE (nm)	*f*
$S_0 - S_1$	90.3	316.5	0.135	97.3	293.8	0.078
$S_0 - S_2$	137.1	208.6	0.054	129.1	221.5	0.031

2 Excited State Lifetime

We calculated the excited state lifetime (τ) by fitting the S_1 population as a function of time using the equations in Figure S2.

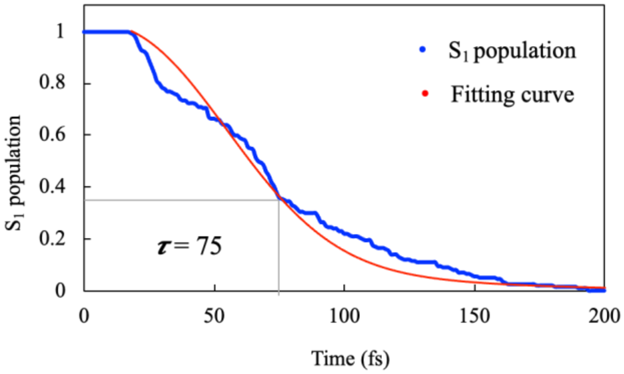

$$\boldsymbol{\tau} = t_1 + a_1 + t_2 + (1 - a_1 - y_0)t_3$$

$$f(x) = a_1 e^{\left(-\left(\frac{t-t_1}{t_2}\right)^2\right)} + y_0 + ((1 - a_1) - y_0) e^{\left(-\left(\frac{t-t_1}{t_3}\right)\right)}$$

a_1 = weight of gaussian decay component
y_0 = fitting parameter
t_1 = latency time
t_2 = decay time of gaussian component
t_3 = decay time of exponential component

Figure S2. The predicted excited state lifetime of 1 in MeCN is obtained using quantum-classical dynamics. The blue and red lines in the plot represent the S_1 population fractions and the fitted population, respectively. The equation determining the excited state lifetime τ of the S_1 population vs. time is provided. The fitting model *f(x)* used to determine the parameters t_1, t_2, t_3, and a_1 is also given.

3 Boltzmann Sampling Distribution

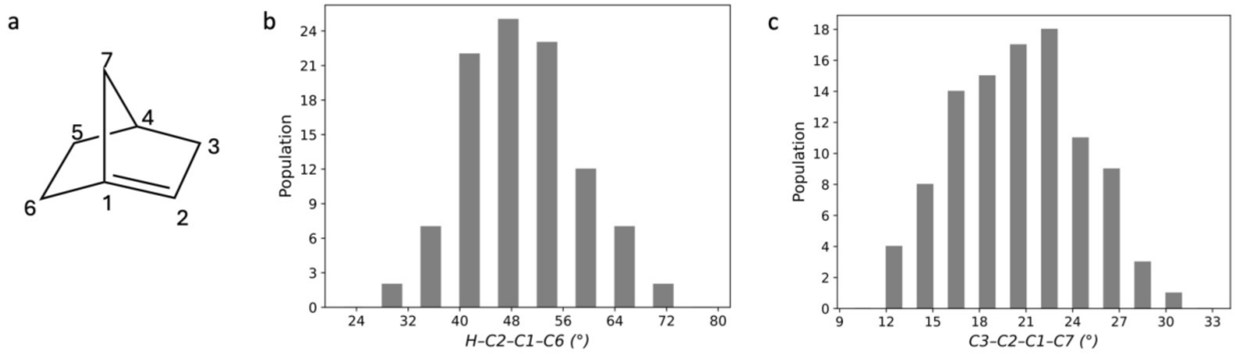

Figure S3. Boltzmann sampling distribution of 1 in MeCN. a) Conventional numerical labeling of **1**. b) Dihedral angle distribution representing the position of the H atom at C2. This is found to assume two equilibrium positions (leading to two populations) at room temperature. The excess population (with respect to a Gaussian distribution) around 60º contributes to a shoulder (near 400 nm) in the predicted absorption spectrum (see Figure 1c). c) Dihedral angle distribution representing the variation in skeletal double bond torsion.

4 Geometrical and Electronic Structure Analyses

4.1 CoIn Topography along the Branching Plane

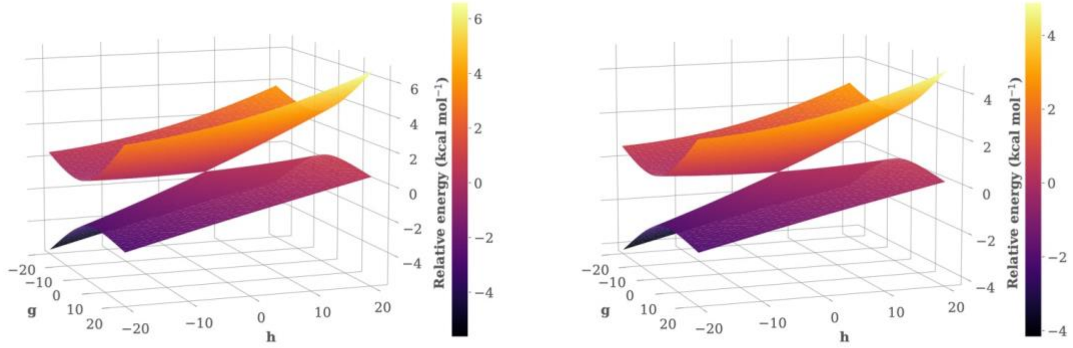

Figure S4. 3D representation of the S_0 and S_1 PES plotted along the branching plane. The plot is generated by computing a grid (10^{-3} Å x 10^{-3} Å) along the plane defined by the branching plane vectors (g and h) of **1** obtained at both the SA3 CASSCF/aug-cc-pVDZ (left) and SA3 RMS-CASPT2/aug-cc-pVDZ (right) levels of theory for CAS(4,4). Notice the difference in the energy gap. At the RMS-CASPT2 level,

the S_1-S_0 energy gap is lower with respect to the CASSCF level. However, the topography is similar at the two levels.

4.1 Molecular Geometry Analysis

In Table S2, we compare the values of different torsional angles for the S_0 equilibrium structure *in vacuo*. The values for a representative S_0 equilibrium structure in solution can be found in Figure 1d. The table shows that the values calculated at the SA3 RMS-CASPT2/aug-cc-pVDZ and SA3 RMS-CASPT2/6-31+G* levels are generally comparable to those calculated at the MP2 level and DFT level, especially when using the ωB97XD functional.

Table S2. Dihedral angles for the equilibrium structure of 1 *in vacuo*. Comparison of the critical dihedral angles and bond lengths describing the double bond geometry in the S_0 equilibrium geometry of **1** computed at the SA3 RMS-CASPT2/aug-cc-pVDZ and SA3 RMS-CASPT2/6-31+G* levels (present work) and computed with a variety of other methods (see Ref. entry for the reference) including HF, MP2, and DFT methods.

Level of Theory	Torsion (H-C2-C1-C6) (°)	Torsion (C3-C2-C1-C7) (°)	Twist angle (°)	C1-C2 (Å)	Ref.
RMS-CASPT2/6-31+G*	50.9	21.6	36.3	1.38	Present Work
RMS-CASPT2/aug-cc-pVDZ	48.7	21.6	35.2	1.39	Present Work
UFF	64.4	34.9	49.7	1.34	L. McDermott et al., Science 386, eadq3519 (2024)
PM6	72.1	27.2	49.7	1.37	L. McDermott et al., Science 386, eadq3519 (2024)
HF/6-31G*	55.6	22.1	38.9	1.35	L. McDermott et al., Science 386, eadq3519 (2024)
B3LYP/6-31G*	51.4	22.0	36.8	1.37	L. McDermott et al., Science 386, eadq3519 (2024)
B3LYP-D3/6-311+G(d,p)	49.9	22.2	36.1	1.36	L. McDermott et al., Science 386, eadq3519 (2024)
B3PW91/6-31G*	51.5	21.9	36.7	1.36	L. McDermott et al., Science 386, eadq3519 (2024)
MP2/6-311+G(d,p)	49.0	20.6	35.0	1.38	L. McDermott et al., Science 386, eadq3519 (2024)
ωB97XD/def2-TZVP	49.3	22.2	35.7	1.35	L. McDermott et al., Science 386, eadq3519 (2024)

In Figure S5, we display the optimized geometries of representative S_0 equilibrium structures ($S_{0\text{-}min}$), S_1 equilibrium structures ($S_{1\text{-}min}$), minimum energy S_1/S_0 conical intersections (CoIn), and related photoproducts (also energy minima on S_0) with the relevant bond lengths. A photoproduct (a minimum on the S_0 PES) with zwitterionic rather than diradical character could not be located. Additionally, with the expanded active space orbitals (i.e., with the inclusion of C1-C7 sigma orbitals), we report a new S_1 minimum, named $S_{1\text{-}min}$ (C1-C7), and a new CoIn, named CoIn (C1-C7), describing the breaking of the C1-C7 bond. The energies of these structures indicate that breaking the C1-C7 bond is energetically unfavorable on the S_1 PES.

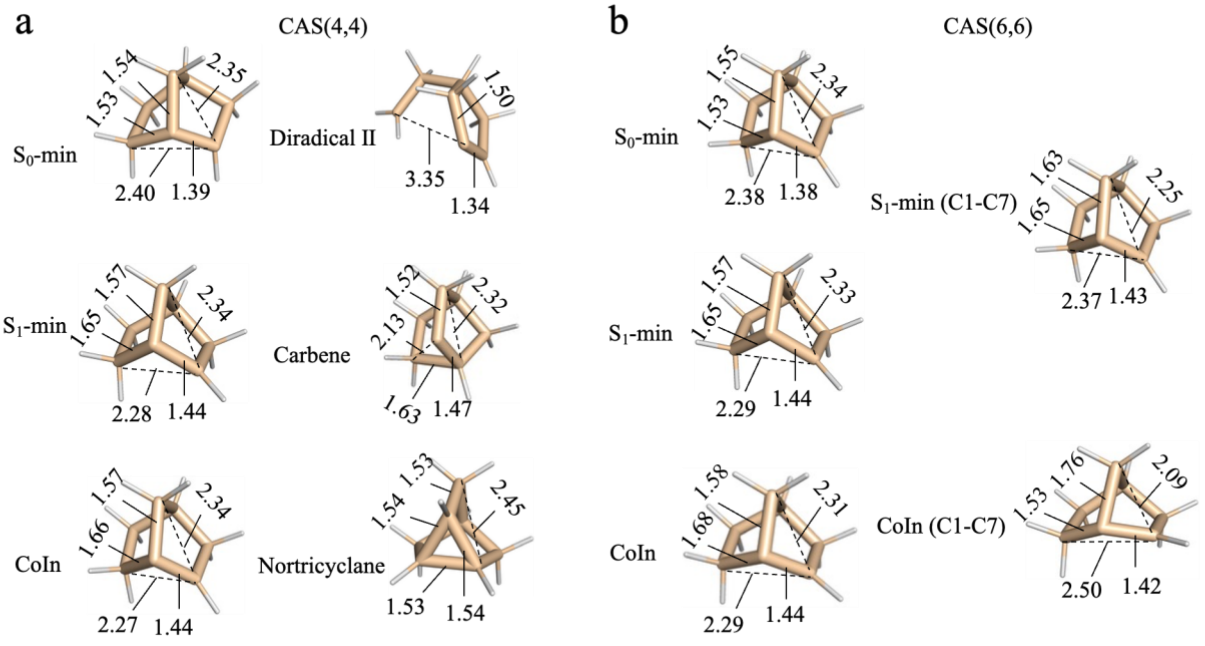

Figure S5. Optimized geometries of $S_{0\text{-min}}$, $S_{1\text{-min}}$, CoIn, and photoproducts in MeCN. a) Optimized geometries of $S_{0\text{-min}}$, $S_{1\text{-min}}$, CoIn, and photoproducts (also S_0 PES minima) Diradical II, Carbene, and Nortricyclane computed at the CAS(4,4) level. b) Optimized geometries of $S_{0\text{-min}}$, $S_{1\text{-min}}$, CoIn, and photoproducts computed at the CAS(6,6) expanded level to include the C1-C7 sigma bond orbitals. All bond length values are shown in Å. Tint colored atoms correspond to C, and white atoms correspond to H.

In Table S3 below, we show the corresponding energy data providing evidence for a limited effect of the expansion from CAS(4,4) to CAS(6,6). For instance, if we look at the relative energy of the CoIn, the difference is just ~0.1 kcal/mol. The results in Table S3 suggest that, to save computer time, the expansion of the active space orbitals can be avoided when assuming, as suggested by the energy analysis, a low efficiency for the C1-C7 bond breaking process.

Table S3. The potential energies for the optimized geometries. Potential energy comparison for optimized geometries of S_0, S_1, CoIn, and the photoproducts computed at the SA3 RMS-CASPT2/aug-cc-pVDZ.

CAS(4,4)						
Geom. Opt. Type	$S_{0\text{-min}}$	$S_{1\text{-min}}$	CoIn	Carbene	Nortricyclane	Diradical II
S_0 (Hartree)	-272.16081899	-272.09141581	-272.08307282	-272.12614819	-272.22911970	-272.09881023
S_1 (Hartree)	-272.01176399	-272.08379796	-272.08187354	-272.02615688	-271.98710649	-271.99952649
S_0 Ediff rel. to S_0opt (kcal/mol)	0.0	43.6	48.8	21.8	-42.9	38.9
S_1 Ediff rel. to S_0opt (kcal/mol)	93.5	48.3	49.5	84.5	109.0	101.2
Egap (kcal/mol)	93.5	4.8	0.8	62.7	151.9	62.3
CAS(6,6)						
Geom. Opt. Type	$S_{0\text{-min}}$	$S_{1\text{-min}}$	CoIn	$S_{1\text{-min}}$ (C-C7)	CoIn (C-C7)	
S_0 (Hartree)	-272.15936441	-272.08935053	-272.08173452	-272.08327169	-272.06432722	
S_1 (Hartree)	-272.00896992	-272.08211042	-272.08111879	-272.07004736	-272.05298430	
S_0 Ediff rel. to S_0opt (kcal/mol)	0.0	43.9	48.7	47.7	59.6	
S_1 Ediff rel. to S_0opt (kcal/mol)	94.4	48.5	49.1	56.0	66.8	
Egap (kcal/mol)	94.4	4.5	0.4	8.3	7.1	

4.2 Electronic Structure (Bonding) Analysis

In this subsection, we report the results related to establishing the electronic structure (character) of the S_1 and S_0 states of the different optimized structures in solution. This is to define the correct nature of the products "generated" at the end of the analyzed trajectories (see Figure 3c). Such products are Diradical I (ring opening with a relatively short bond distance of C1-C6, >1.7 Å) and Diradical II (ring opening with a relatively long bond distance of C1-C6, >2.5 Å). When starting a geometry optimization searching for an S_0 minimum from such points, one obtains either the reactant (for the "unstable" Diradical I) or a stable diradical (Diradical II), respectively. We concluded that Diradical I is transient and does not lead to a minimum on the S_0 PES. The same test for the carbene "product" also yields an S_0 minimum with diradical character. However, through this analysis, it has been impossible to locate (as an S_0 minimum) a stable intermediate with zwitterionic character. We conclude that such an entity has a transient existence and does not correspond to a minimum on the S_0 PES (see Figures 2 and 3).

As discussed in the main text, we determine the electronic character of the reactant, photoproducts, transient S_1 and S_0 structures, as well as structures (points) in the CoIn branching plane via either charge analysis (Mulliken and Hirshfeld) or using Mayer free valence (FV) and bond order (BO) analyses. More precisely, we rely on the consistency of the results from the charge analyses and Mayer analysis to assign the character of the states (e.g. S_1 or S_0) of a specific geometrical structure. It is found that, irrespective of significant differences in the charge values, the Mulliken and Hirshfeld analyses produce the same qualitative information relative to the fragments I, II, and III of **1** defined in the main text. These results are given in Tables S4, S5, and S6.

Table S4. Mulliken and Atomic Dipole Corrected Hirshfeld (ADCH) charges analysis. For comparison purposes, below we provide two different charges for the optimized geometries of $S_{0\text{-min}}$, $S_{1\text{-min}}$, CoIn, and the photoproducts using Multiwfn[16,17] analyzer software.

CAS (4,4)						
	$S_{0\text{-min}}$		$S_{1\text{-min}}$		CoIn	
Fragments	Mulliken	ADCH	Mulliken	ADCH	Mulliken	ADCH
I	0.35	0.07	0.45	0.22	0.53	0.25
II	-0.85	-0.06	-1.14	-0.62	-1.21	-0.65
III	0.50	-0.01	0.69	0.40	0.67	0.40
	Carbene		Nortricyclane		Diradical II	
Fragments	Mulliken	ADCH	Mulliken	ADCH	Mulliken	ADCH
I	0.34	0.09	-0.18	0.21	0.29	-0.02
II	-0.81	-0.26	0.18	-0.19	-0.66	0.02
III	0.47	0.17	0.00	-0.01	0.37	0.00
CAS (6,6)						
	$S_{0\text{-min}}$		$S_{1\text{-min}}$		CoIn	
Fragments	Mulliken	ADCH	Mulliken	ADCH	Mulliken	ADCH
I	0.32	0.07	0.25	0.11	0.25	0.11
II	-0.76	-0.07	-0.93	-0.49	-0.99	-0.55
III	0.43	0.00	0.67	0.38	0.73	0.44
	$S_{1\text{-min}}$ (C1-C7)		CoIn (C1-C7)			
Fragments	Mulliken	ADCH	Mulliken	ADCH		
I	0.25	0.11	0.41	0.15		
II	-0.93	-0.49	-1.12	-0.45		
III	0.67	0.38	0.71	0.29		

Table S5. Free valence analysis. As part of the electronic structure analysis, we report the FV value (i.e., free valences, namely the number of unpaired electrons) at the optimized geometries of $S_{0\text{-min}}$, $S_{1\text{-min}}$, CoIn, photoproducts, and $S_{1\text{-min}}$ (C1-C7) and CoIn (C1-C7). To do so, we use the Multiwfn[16,17] analyzer software.

CAS (4,4)						
	$S_{0\text{-min}}$	$S_{1\text{-min}}$	CoIn	Carbene	Nortricyclane	Diradical II
	Free valences	Free valences	Free valences	Free valences	Free valences	Free valences
C1	0.38	0.08	0.05	0.10	0.05	1.16
C2	0.26	0.13	0.10	0.04	0.05	0.15
C3	0.01	0.00	0.00	0.04	0.00	0.02
C4	0.01	0.01	0.00	0.00	0.00	0.03
C5	0.01	0.00	0.00	0.00	0.00	0.00
C6	0.02	0.03	0.03	0.00	0.09	0.97
C7	0.01	0.01	0.01	0.00	0.00	0.00
CAS (6,6)						
	$S_{0\text{-min}}$	$S_{1\text{-min}}$	CoIn	$S_{1\text{-min}}$ (C1-C7)	CoIn (C1-C7)	
C1	0.38	0.24	0.18	0.24	0.14	
C2	0.31	0.19	0.14	0.19	0.15	
C3	0.01	0.00	0.00	0.00	0.00	
C4	0.01	0.01	0.01	0.01	0.00	
C5	0.01	0.01	-0.01	0.01	0.00	
C6	0.00	0.08	0.08	0.08	0.00	
C7	0.04	0.01	0.01	0.01	0.11	

The BO analysis has focused on the bond lengths describing the main reaction coordinates: C1-C6, C2-C6, C1-C7, and C1-C2. For instance, if we take the geometry for carbene, C1-C6 has a BO of 0.11 (no bond or the bond is broken), C2-C6 has a BO of 1.04 (single bond, formation of a new C-C bond), C1-C7 has a BO of 1.20 (single bond, no change), and C1-C2 has a BO of 0.83 (change of double bond to single bond).

Table S6. Bond order analysis. We perform the BO (i.e., bond order) analysis for the optimized geometries $S_{0\text{-min}}$, $S_{1\text{-min}}$, CoIn, photoproducts, and $S_{1\text{-min}}$ (C1-C7) and CoIn (C1-C7). To do so, we use the Mayer analysis via Multiwfn[16,17] analyzer software.

Bonds	$S_{0\text{-min}}$	$S_{1\text{-min}}$	CoIn	Carbene	Nortricyclane	Diradical II	$S_{0\text{-min}}$ CAS (6,6)	$S_{1\text{-min}}$ CAS (6,6)	CoIn CAS (6,6)	$S_{1\text{-min}}$ (C1-C7)	CoIn (C1-C7)
C1-C6	1.13	0.95	0.85	0.11	0.85	0.00	1.12	0.88	0.87	0.88	1.06
C2-C6	0.07	0.11	0.18	1.04	0.85	0.00	0.09	0.08	0.10	0.08	0.00
C1-C7	0.99	1.11	1.02	1.20	1.22	1.16	0.96	1.07	1.06	1.07	0.68
C1-C2	1.52	1.23	1.08	0.83	0.91	1.61	1.49	1.20	1.16	1.20	0.87

4.4. Is S_2 a Rydberg or valence state?

In Table S7, we examine the order of the states of **1** by expanding the adopted CAS(4,4) with two Rydberg orbitals (this leads to a CAS(4,6) different from the one seen above to account for the possible C1-C7 bond breaking) and determine whether a specific S_n is a Rydberg state or not. We find that, regardless of the expansion of the active space, S_1 is always a bright valence state. The expectation value ($\langle r^2 \rangle$) as well as wave function analysis show that when $n \geq 2$, the states have either Rydberg or valence characters, with the Rydberg character dominating S_2 and S_3 and the valence character dominating S_4. The computation was performed at the SA5 RMS-CASPT2/aug-cc-pVDZ level using OpenMolcas-v24.06.

Table S7. Expectation value analysis (2nd Cartesian moment) and wave function analysis. Vertical excitation energies and oscillator strengths (f) of each of the five excited singlet states (S_0-S_4). In the presented table, expectation values of excited states were calculated relative to $\langle r^2 \rangle S_0$. Note: the present wavefunctions are RMS-CASPT2, not CASSCF level. Molecular orbitals were plotted using the Pegamoid software package with an isosurface value of 0.02.

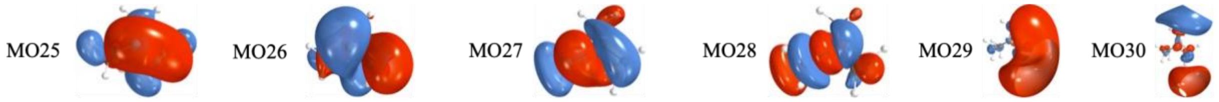

Expectation value analysis (2-nd cartesian moments)

States	VEE (nm)	f	$\langle x^2 \rangle$ (a.u.^{2})	$\langle y^2 \rangle$ (a.u.^{2})	$\langle z^2 \rangle$ (a.u.^{2})	$\langle r^2 \rangle$ (a.u.^{2})	$\Delta\langle r^2 \rangle_{rel}$ (a.u.^{2})	Character
S_0			-35.30	-30.54	-30.50	-96.33	0.00	GS
S_1	324.8	0.128	-69.16	-48.88	-44.85	-162.88	-66.55	π-π*/Valence
S_2	231.8	0.030	-45.42	-53.77	-43.16	-142.35	-46.02	π-3S/Rydberg
S_3	212.4	0.012	-42.57	-41.51	-34.56	-118.64	-22.30	π-3Py/Rydberg
S_4	203.2	0.019	-41.18	-31.38	-33.39	-105.95	-9.62	π-π*/Valence

Wave function analysis (occupation number and weight)

State	Occ.	Coefficient	Weight	State	Occ.	Coefficient	Weight	State	Occ.	Coefficient	Weight	State	Occ.	Coefficient	Weight	State	Occ.	Coefficient	Weight
S_0	u2d000	-0.054173	0.002935	S_1	2ud000	0.073453	0.005395	S_2	20u0d0	0.063695	0.004057	S_3	20u00d	0.061995	0.003843	S_4	220000	0.130599	0.017056
	202000	-0.962089	0.925616		u2d000	-0.098475	0.009697		udu0d0	0.078646	0.006185		udu00d	0.078444	0.006154		2ud000	0.357301	0.127664
	22000	0.230784	0.053261		ud2000	0.980993	0.962348		u020d0	0.980826	0.962021		u020d0	-0.074411	0.005537		u2d000	-0.076581	0.005865
	20020	0.076248	0.005814		20ud00	0.061607	0.003795		u0200d	0.070977	0.005038		u0200d	0.979417	0.959258		202000	-0.215992	0.046652
					ud0200	-0.073185	0.005356		0u200d	-0.069833	0.004877		0u20d0	-0.088088	0.007759		ud2000	-0.052509	0.002757
									u002d0	-0.080431	0.006469		u0020d	-0.079816	0.006371		22000	-0.883418	0.780428

4.5. Extended CoIn analysis and zwitterionic character

To examine the zwitterionic character on the S_0 PES of 1 (with the hope of locating a stable zwitterionic intermediate), we performed two sets of calculations. In the first set, we selected all trajectories that display a large zwitterionic character (a large negative Mulliken charge of fragment II, namely < -1) at their last point (snapshots; see Figure S6). For each of the eight selected trajectories, we performed an S_0 geometry optimization starting from such a last point with the hope of locating a zwitterionic minimum. However, no zwitterionic minimum was found, as all geometry optimizations ended in either a diradical, carbene, or reactant minimum. The second set of calculations was performed with the objective of studying the extension of the zwitterionic character along the branching plane of the CoIn. The results of this second set of calculations are displayed in Figure 7 (see also Figure 2e). The changes in Mulliken charges of fragments I, II, and III of the CoIn inform about the extension of the zwitterionic character along and beyond the branching plane. It is observed that in the g positive direction, the zwitterionic character is maintained and reinforced. In spite of this, no zwitterionic minimum could be located when starting an S_0 geometry relaxation from points belonging to that region. More specifically, by moving the center of the CoIn circle, also seen in Figure 2e, in the same g direction of 0.02 Å, we generated three additional circles for a total of four circles centered on points 0.0, 0.02, 0.04, and 0.08. We then plotted the S_0 charges of fragments I, II, and III along the four different circles

to explore the zwitterionic character change (see Figure 7a). The increase in the extension of the negative charge along these circular cross-sections indicates a "vast" zwitterionic region in this area. The same results are qualitatively observed with ADCH charges (see Figure 7b). The plot representing the change in geometry along g, and pictorially, the change in the extension of the zwitterionic region relative to the diradical/carbene region, is shown in Figure 7c.

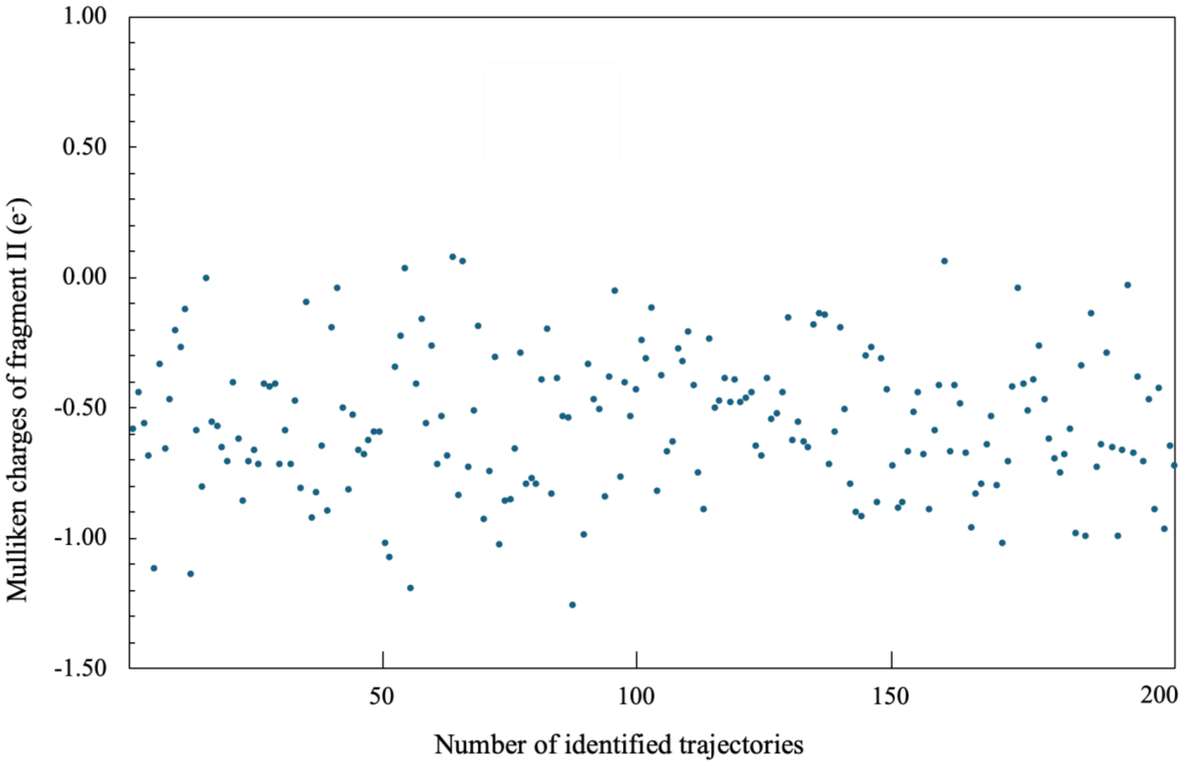

Figure S6. Distribution of the S_0 charge at the last point along each trajectory. Charges at the last point (time = 200 fs) for each of the 200 computed quantum-classical trajectories. The abscissa values identify the individual trajectories. The points with charges less than -1 are the ones tested via geometry optimization on S_0.

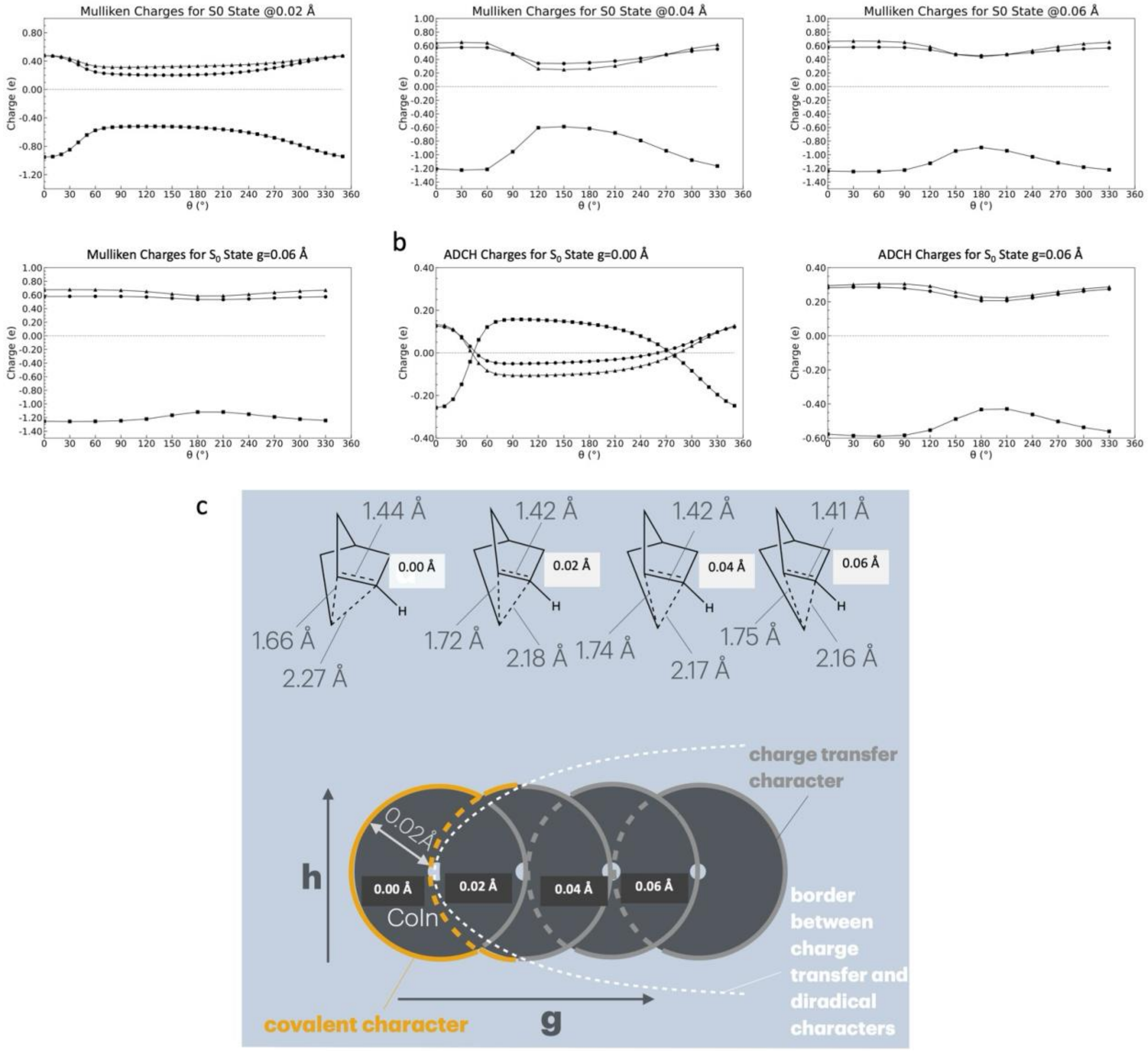

Figure S7. Fragments I, II, and III S_0 charge along different circular (0.02 Å radius) cross-sections located at different points of the branching vector g. a) Lines of circles, squares, and triangles filled with black color represent fragments I, II, and III (see main text for fragment details) of **1**, respectively. The g values (0.00, 0.02, 0.04, and 0.06 Å) represent the positions of the centers of the circles relative to the CoIn center. b) Same diagrams but for just two circles (g=0.00 and g=0.06 Å) when using ADCH charges. c) Change in geometry and schematic representation of the four circles. When considering the information provided in parts a and b, it is evident that the zwitterionic region extends significantly along the g direction.

5 Cartesian Coordinates

Cartesian coordinates of the optimized geometries of $S_{0\text{-min}}$, $S_{1\text{-min}}$, CoIn, and the photoproducts.

$S_{0\text{-min}}$ coordinate

17

C	26.09290348	27.36236891	27.41136236
C	27.12548470	27.35203843	26.18867692
C	26.85317952	26.02232950	25.38168016
C	25.27832120	26.16186626	26.91988171
C	24.60248583	26.47935524	25.74684832
C	25.50602662	26.10996300	24.57237370
C	26.35686065	25.13033030	26.53554664
H	25.52018925	28.30119639	27.44894722
H	26.59317948	27.19112180	28.37789447
H	28.16874804	27.33860073	26.54955431
H	26.99284428	28.24275598	25.55230567
H	27.72309666	25.68094214	24.79464231
H	25.48800214	26.90985997	23.81173977
H	25.31058860	25.15433402	24.05284465
H	27.10589487	24.97477496	27.32893369
H	25.94784751	24.16324954	26.20563733
H	23.94536555	27.35589750	25.70138467

$S_{1\text{-min}}$ coordinate

17

C	25.76088887	27.41501484	26.93089981
C	26.93643517	27.50414333	25.93624890
C	26.80843647	26.13043683	25.26154750
C	25.25300087	25.83208055	27.02638225
C	24.49879995	25.90267398	25.80277222
C	25.40623011	26.13223055	24.61812209
C	26.59053816	25.20644955	26.51022434
H	24.93454463	28.11138919	26.71780723
H	26.04056681	27.55062537	27.99123071

H	27.90708002	27.59997528	26.45018282
H	26.83333292	28.33439334	25.22083823
H	27.62594468	25.85135156	24.58016168
H	25.18807475	26.98305353	23.94872673
H	25.24233448	25.20463453	24.02612420
H	27.41220109	25.29736944	27.23941380
H	26.46717491	24.14663119	26.23331141
H	23.43978628	25.64816811	25.65949311

CoIn coordinate

17

C	26.31586687	26.78332724	26.23558103
C	27.27831370	26.94837385	25.04324483
C	26.96285032	25.63866964	24.30585800
C	25.75829044	25.21771617	26.29669423
C	24.78571737	25.42979689	25.25541621
C	25.46694429	25.73465664	23.94636666
C	26.96416614	24.61498811	25.49792408
H	25.50229496	27.52500895	26.26565505
H	26.79832529	26.78032643	27.23474218
H	28.33549776	26.98726639	25.35051956
H	27.06524965	27.84067188	24.43597816
H	27.62042672	25.39952797	23.45692646
H	25.16716690	26.61173761	23.34935415
H	25.15754906	24.82705724	23.37666336
H	27.89282684	24.66994200	26.08764148
H	26.76665665	23.57840884	25.17497950
H	23.71246202	25.19586859	25.29325898

Carbene coordinate

17

C	28.18393936	24.76423892	25.56450035
C	26.72240058	24.25755112	25.49470477
C	25.95009508	25.52909231	25.90221220
C	27.99621190	26.30752053	27.02906007
C	28.04010267	26.38314828	25.55929197
C	26.61560810	26.57689416	24.99245215
C	26.54036250	25.94046624	27.27094699
H	28.82749594	24.51169601	24.71067077
H	28.69461660	24.39649412	26.47425019
H	26.54282452	23.41224140	26.17552103
H	26.44933737	23.95031876	24.47223896
H	24.85479937	25.44687930	25.86592820
H	26.53899628	26.34264155	23.91810571
H	26.25130891	27.60309087	25.16910903
H	26.36852552	25.24251977	28.10390759
H	26.12069656	26.92326086	27.58863537
H	28.88503110	26.88344463	25.07263074

Nortricyclane coordinate

17

C	25.71238997	23.46683778	27.48593563
C	26.63298993	24.66482967	27.70623712
C	26.05782350	25.55148803	26.57134179
C	25.40229168	23.41412982	25.98723330
C	24.38003936	24.01404266	26.96323810
C	24.54104316	25.53142846	26.88619276
C	26.14031064	24.59476523	25.35488925
H	25.75698507	22.56135237	28.09432697
H	25.23397535	22.46594859	25.46955174
H	27.69407972	24.41679560	27.54430079
H	26.51463446	25.11534684	28.70539267

H	26.51189433	26.54422276	26.44355687
H	24.30169019	26.03749122	27.83615337
H	23.94550509	25.99432883	26.07947145
H	27.18046805	24.35199106	25.08076406
H	25.62452756	25.00203943	24.46775639
H	23.43130390	23.52167090	27.19149682

Diradical II coordinate

17

C	26.03560531	27.66067501	26.73302294
C	26.80622782	27.07893867	25.58668622
C	26.32299975	25.68567281	25.15357934
C	24.96333760	24.48676063	26.68405437
C	24.06566156	24.97771726	25.81210389
C	24.82360041	25.70883987	24.70262761
C	26.41486275	24.66069062	26.34412521
H	26.10447826	28.72268985	26.97115763
H	25.42225917	27.02899221	27.37782953
H	27.88334136	27.00397692	25.84932138
H	26.76246461	27.76434421	24.71925882
H	26.96282078	25.34144535	24.32644545
H	24.47202242	26.74725828	24.56427539
H	24.68453900	25.18453750	23.74210785
H	27.01937378	25.05823794	27.17751889
H	26.86257046	23.70550323	26.02565726
H	22.98047962	24.85103561	25.84094192

$S_{0\text{-min}}$ CAS(6,6) coordinate

17

C	25.83693786	27.26950344	27.56374116
C	26.94059905	27.54238418	26.43317397

C	26.95975177	26.25613269	25.51620738
C	25.27702959	25.99317678	26.93369492
C	24.65813173	26.29802816	25.73431525
C	25.68016673	26.17255683	24.60462488
C	26.55141852	25.20238267	26.56285382
H	25.10783766	28.09120468	27.61854515
H	26.29084905	27.11119669	28.55307618
H	27.94615413	27.67767723	26.86675675
H	26.69737125	28.44983459	25.85589376
H	27.91476713	26.11833774	24.98024708
H	25.56672396	27.01825171	23.90293338
H	25.69420440	25.24416242	24.00617854
H	27.25877267	25.11019349	27.40220431
H	26.34493461	24.20881829	26.13800529
H	23.84480301	27.03125770	25.68738285

$S_{1\text{-min}}$ CAS(6,6) coordinate

17

C	26.43264269	26.72130677	26.11543877
C	27.38538216	26.90215987	24.91201338
C	27.10708552	25.58927089	24.16445276
C	25.90254475	25.15593686	26.15135255
C	24.92957502	25.32906984	25.10176919
C	25.61118211	25.63908623	23.79672798
C	27.11592272	24.56242347	25.35108967
H	25.60354531	27.44547860	26.14119195
H	26.93113213	26.74961896	27.10349755
H	28.44129957	26.96354418	25.22109803
H	27.15333338	27.79157307	24.30698645
H	27.77326522	25.37054505	23.31601355
H	25.27980533	26.51686489	23.21774973

H	25.32794774	24.73633894	23.20978229
H	28.04259075	24.61954742	25.94379737
H	26.92501075	23.52534547	25.02617698
H	23.85961651	25.08813632	25.13895472

CoIn CAS(6,6) coordinate

17

C	26.40686479	26.75353598	26.29003526
C	27.34682117	26.93131482	25.08221406
C	27.04144091	25.62235903	24.34007112
C	25.86292893	25.16174632	26.37042170
C	24.89314763	25.33101580	25.31815723
C	25.53871402	25.68984416	24.00660223
C	27.07169622	24.59322596	25.51978033
H	25.57691518	27.47656265	26.32301950
H	26.91310251	26.77388682	27.27076743
H	28.40759857	26.97056954	25.38107492
H	27.12365659	27.82475807	24.47925000
H	27.68706926	25.40262155	23.47636793
H	25.20714195	26.59070446	23.46265411
H	25.23623924	24.81726705	23.38829159
H	28.00639363	24.66079991	26.10323758
H	26.88800127	23.55282394	25.20182521
H	23.82949971	25.06217175	25.36180874

$S_{1\text{-min}}$ (C1-C7) coordinate

17

C	25.40104902	26.89385295	25.56837079
C	25.13730949	25.36577622	25.55510356
C	26.57556617	24.84012204	25.45002174
C	26.99547280	27.22902795	25.34503260

C	27.55232989	26.69338920	26.54222861
C	27.27641915	25.22121187	26.76699793
C	27.22383813	25.87574668	24.46919499
H	25.03216113	27.40822682	26.46770141
H	24.96472960	27.39053083	24.68554567
H	24.53368451	25.05721337	24.68758053
H	24.63310468	25.02513313	26.47519556
H	26.68777588	23.78070172	25.17068555
H	26.75535478	24.96066604	27.70758025
H	28.29007747	24.76541105	26.81759825
H	26.67485311	25.97647447	23.52373073
H	28.28648998	25.66060036	24.26709364
H	28.30628991	27.19322757	27.17435810

CoIn (C1-C7) coordinate

17

C	24.37613460	26.22834200	25.39343852
C	24.83119004	24.73506723	25.45641878
C	26.34494964	24.78770001	25.21374734
C	25.59852030	27.13800213	25.24734910
C	26.66615657	26.88721366	26.15249796
C	26.99416813	25.41553616	26.46725256
C	26.51478401	26.00227118	24.27051152
H	23.82356138	26.53010457	26.29179397
H	23.69551277	26.35735938	24.54329850
H	24.31229731	24.17616463	24.66677902
H	24.61228045	24.28354596	26.43683097
H	26.79987105	23.85213025	24.85269071
H	26.53377739	25.08292644	27.41540868
H	28.08639333	25.25667933	26.52171939
H	25.88525492	26.03907565	23.36630184

H	27.55006254	26.28351878	24.03327124
H	27.39998849	27.66487263	26.39610200

7 References

(1) Frisch, M. J.; Head-Gordon, M.; Pople, J. A. A Direct MP2 Gradient Method. *Chem. Phys. Lett.* **1990**, 166 (3), 275–280.

(2) Head-Gordon, M.; Pople, J. A.; Frisch, M. J. MP2 Energy Evaluation by Direct Methods. *Chem. Phys. Lett.* **1988**, 153 (6), 503–506.

(3) Miertuš, S.; Scrocco, E.; Tomasi, J. Electrostatic Interaction of a Solute with a Continuum: A Direct Utilization of ab initio Molecular Potentials for the Prediction of Solvent Effects. *Chem. Phys.* **1981**, 55 (1), 117–129.

(4) Frisch, M. J.; Trucks, G. W.; Schlegel, H. B.; Scuseria, G. E.; Robb, M. A.; Cheeseman, J. R.; Scalmani, G.; Barone, V.; Petersson, G. A.; Nakatsuji, H.; Li, X.; Caricato, M.; Marenich, A. V.; Bloino, J.; Janesko, B. G.; Gomperts, R.; Mennucci, B.; Hratchian, H. P.; Ortiz, J. V.; Izmaylov, A. F.; Sonnenberg, J. L.; Williams-Young, D.; Ding, F.; Lipparini, F.; Egidi, F.; Goings, J.; Peng, B.; Petrone, A.; Henderson, T.; Ranasinghe, D.; Zakrzewski, V. G.; Gao, J.; Rega, N.; Zheng, G.; Liang, W.; Hada, M.; Ehara, M.; Toyota, K.; Fukuda, R.; Hasegawa, J.; Ishida, M.; Nakajima, T.; Honda, Y.; Kitao, O.; Nakai, H.; Vreven, T.; Throssell, K.; Montgomery, J. A., Jr.; Peralta, J. E.; Ogliaro, F.; Bearpark, M.; Heyd, J. J.; Brothers, E.; Kudin, K. N.; Staroverov, V. N.; Kobayashi, R.; Normand, J.; Raghavachari, K.; Rendell, A.; Burant, J. C.; Iyengar, S. S.; Tomasi, J.; Cossi, M.; Millam, J. M.; Klene, M.; Adamo, C.; Cammi, R.; Ochterski, J. W.; Martin, R. L.; Morokuma, K.; Farkas, O.; Foresman, J. B.; Fox, D. J. Gaussian 09, Revision A.01; Gaussian, Inc.: Wallingford, CT, USA, **2016**.

(5) Caleman, C.; van Maaren, P. J.; Hong, M.; Hub, J. S.; Costa, L. T.; van der Spoel, D. Force Field Benchmark of Organic Liquids: Density, Enthalpy of Vaporization, Heat Capacities, Surface Tension, Isothermal Compressibility, Volumetric Expansion Coefficient, and Dielectric Constant. *J. Chem. Theory Comput.* **2012**, 8 (1), 61–74.

(6) Dodda, L. S.; Cabeza de Vaca, I.; Tirado-Rives, J.; Jorgensen, W. L. LigParGen Web Server: An Automatic OPLS-AA Parameter Generator for Organic Ligands. *Nucleic Acids Res.* **2017**, 45 (W1), W331–W336.

(7) Jorgensen, W. L.; Tirado-Rives, J. Potential Energy Functions for Atomic-Level Simulations of Water and Organic and Biomolecular Systems. *Proc. Natl. Acad. Sci. U. S. A.* **2005**, *102* (19), 6665–6670.

(8) Dodda, L. S.; Vilseck, J. Z.; Tirado-Rives, J.; Jorgensen, W. L. 1.14*CM1A-LBCC: Localized Bond-Charge Corrected CM1A Charges for Condensed-Phase Simulations. *J. Phys. Chem. B* **2017**, 121 (15), 3864–3870.

(9) Pronk, S.; Páll, S.; Schulz, R.; Larsson, P.; Bjelkmar, P.; Apostolov, R.; Shirts, M. R.; Smith, J. C.; Kasson, P. M.; van der Spoel, D.; Hess, B.; Lindahl, E. GROMACS 4.5: A High-Throughput and Highly Parallel Open Source Molecular Simulation Toolkit. *Bioinformatics* **2013**, 29 (7), 845–854.

(10) Boström, J.; Veryazov, V.; Aquilante, F.; Pedersen, T. B.; Lindh, R. Analytical Gradients of the Second-Order Møller–Plesset Energy Using Cholesky Decompositions. *Int. J. Quantum Chem.* **2014**, 114 (5), 321–327.

(11) Nishimoto, Y.; Battaglia, S.; Lindh, R. Analytic First-Order Derivatives of (X)MS, XDW, and RMS Variants of the CASPT2 and RASPT2 Methods. *J. Chem. Theory Comput.* **2022**, *18* (7), 4269–4281.

(12) Fdez. Galván, I.; Vacher, M.; Alavi, A.; Angeli, C.; Aquilante, F.; Autschbach, J.; Bao, J. J.; Bokarev, S. I.; Bogdanov, N. A.; Carlson, R. K.; Chibotaru, L. F.; Creutzberg, J.; Dattani, N.; Delcey, M. G.; Dong, S. S.; Dreuw, A.; Freitag, L.; Frutos, L. M.; Gagliardi, L.; Gendron, F.; Giussani, A.; González, L.; Grell, G.; Guo, M.; Hoyer, C. E.; Johansson, M.; Keller, S.; Knecht, S.; Kovačević, G.; Källman, E.; Li Manni, G.; Lundberg, M.; Ma, Y.; Mai, S.; Malhado, J. P.; Malmqvist, P. Å.; Marquetand, P.; Mewes, S. A.; Norell, J.; Olivucci, M.; Oppel, M.; Phung, Q. M.; Pierloot, K.; Plasser, F.; Reiher, M.; Sand, A. M.; Schapiro, I.; Sharma, P.; Stein, C. J.; Sørensen, L. K.; Truhlar, D. G.; Ugandi, M.; Ungur, L.; Valentini, A.; Vancoillie, S.; Veryazov, V.; Weser, O.; Wesołowski, T. A.; Widmark, P.-O.; Wouters, S.; Zech, A.; Zobel, J. P. OpenMolcas: From Source Code to Insight. *J. Chem. Theory Comput.* **2019**, 15 (11), 5925–5964.

(13) Li Manni, G.; Fdez. Galván, I.; Alavi, A.; Aleotti, F.; Aquilante, F.; Autschbach, J.; Avagliano, D.; Baiardi, A.; Bao, J. J.; Battaglia, S.; Birnoschi, L.; Blanco-González, A.; Bokarev, S. I.; Broer, R.; Cacciari, R.; Calio, P. B.; Carlson, R. K.; Carvalho Couto, R.; Cerdán, L.; Chibotaru, L. F.; Chilton, N. F.; Church, J. R.; Conti, I.; Coriani, S.; Cuéllar-

Zuquin, J.; Daoud, R. E.; Dattani, N.; Decleva, P.; de Graaf, C.; Delcey, M. G.; De Vico, L.; Dobrautz, W.; Dong, S. S.; Feng, R.; Ferré, N.; Filatov, M.; Gagliardi, L.; Garavelli, M.; González, L.; Guan, Y.; Guo, M.; Hennefarth, M. R.; Hermes, M. R.; Hoyer, C. E.; Huix-Rotllant, M.; Jaiswal, V. K.; Kaiser, A.; Kaliakin, D. S.; Khamesian, M.; King, D. S.; Kochetov, V.; Krośnicki, M.; Kumaar, A. A.; Larsson, E. D.; Lehtola, S.; Lepetit, M. B.; Lischka, H.; López Ríos, P.; Lundberg, M.; Ma, D.; Mai, S.; Marquetand, P.; Merritt, I. C. D.; Montorsi, F.; Mörchen, M.; Nenov, A.; Nguyen, V. H. A.; Nishimoto, Y.; Oakley, M. S.; Olivucci, M.; Oppel, M.; Padula, D.; Pandharkar, R.; Phung, Q. M.; Plasser, F.; Raggi, G.; Rebolini, E.; Reiher, M.; Rivalta, I.; Roca-Sanjuán, D.; Romig, T.; Safari, A. A.; Sánchez-Mansilla, A.; Sand, A. M.; Schapiro, I.; Scott, T. R.; Segarra-Martí, J.; Segatta, F.; Sergentu, D. C.; Sharma, P.; Shepard, R.; Shu, Y.; Staab, J. K.; Straatsma, T. P.; Sørensen, L. K.; Tenorio, B. N. C.; Truhlar, D. G.; Ungur, L.; Vacher, M.; Veryazov, V.; Voß, T. A.; Weser, O.; Wu, D.; Yang, X.; Yarkony, D.; Zhou, C.; Zobel, J. P.; Lindh, R. The OpenMolcas Web: A Community-Driven Approach to Advancing Computational Chemistry. *J. Chem. Theory Comput.* **2023**, *19* (20), 6933–6991.

(14) Sholl, D. S.; Tully, J. C. A Generalized Surface Hopping Method. *J. Chem. Phys.* **1998**, 109 (18), 7702–7710.

(15) Filatov, M.; Paolino, M.; Kaliakin, D.; Olivucci, M.; Kraka, E.; Min, S. K. Impact of Solvation on the Photoisomerisation Dynamics of a Photon-Only Rotary Molecular Motor. *Commun. Phys.* **2024**, 7 (1), 1–9.

(16) Lu, T.; Chen, F. Multiwfn: A Multifunctional Wavefunction Analyzer. *J. Comput. Chem.* **2012**, 33 (5), 580–592.

(17) Lu, T. A Comprehensive Electron Wavefunction Analysis Toolbox for Chemists, Multiwfn. *J. Chem. Phys.* **2024**, 161 (8), 082503.
